# KnowVolution of an Efficient Polyamidase through Molecular Dynamics Simulations of Incrementally Docked Oligomeric Substrates

**DOI:** 10.1002/cssc.202500257

**Published:** 2025-06-26

**Authors:** Hendrik Puetz, Alexander‐Maurice Illig, Mariia Vorobii, Christoph Janknecht, Francisca Contreras, Fabian Flemig, Ulrich Schwaneberg

**Affiliations:** ^1^ Institute of Biotechnology RWTH Aachen University Worringer Weg 3 52074 Aachen Germany; ^2^ DWI‐Leibniz Institute for Interactive Materials Forckenbeckstraße 50 52074 Aachen Germany

**Keywords:** directed evolution, enzymatic polymer degradation, molecular dynamics, nylon, polyamide

## Abstract

Management of synthetic polymer waste is one of the most pressing challenges for society today. Enzymatic recycling of polycondensates like polyamides (PA), however, remains limited due to a lack of efficient polyamidases. This study reports the directed evolution of the polyamidase NylC_p2_–TS. Key positions involved in enzyme–substrate interactions and PA 6 hydrolysis are identified through random mutagenesis and molecular dynamics (MD) simulations. The final variant, NylC–HP (NylC_p2_–TS^F134W/D304M/R330A^), exhibits a 6.9‐fold increased specific activity (520 ± 1 μmol_6–AHAeq_ h^−1^ mg_enzyme_^−1^) and enhanced thermal stability (*T*_m_ = 90 °C, Δ*T*_m_ = 4.2 °C), making NylC–HP the fastest polyamidase for PA 6 and PA 6,6 hydrolysis. Despite the improved reaction rate, the degree of depolymerization remains below 1%. To understand the molecular basis of achieved improvements and factors limiting the degree of depolymerization, intra‐ and intermolecular interactions of various enzyme‐substrate complexes are analyzed by incremental docking of PA 6 tetramers and MD simulations. After optimizing the activity and stability of NylC–HP, the findings suggest that widening the substrate binding pocket is likely necessary to improve substrate accessibility to target more buried attack sites on the polymer surface and thereby enhance the degree of depolymerization.

## Graphical abstract

Managing synthetic polymer waste is a major challenge. While enzymatic polyamide (PA) hydrolysis holds great recycling potential, it is limited by ineffective polyamidases. The polyamidase NylC_p2_‐TS is evolved through random mutagenesis and incremental docking, yielding NylC‐HP with enhanced activity and stability. Molecular dynamics simulations gave insight into the effect of substitutions, advancing the understanding of enzymatic PA recycling.

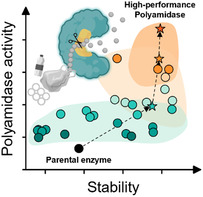

## 1 Introduction

Enzymatic polymer recycling holds the potential to play a pivotal role in the establishment of a circular plastic economy. Consequently, efforts to industrially scale the depolymerization of PET (polyethylene terephthalate) have been actively pursued in recent years. PETases could be applied to completely depolymerize PET at industrially relevant solids loading, bringing cost‐effective PET recycling into reach.^[^ ^1^, ^2^, ^3^ ^]^ This was greatly fueled by enzyme engineering campaigns augmenting the stability, specific activity, and the degree of depolymerization of PETases.^[^ ^2^, ^4^, ^5^ ^]^ Recently, there has been a growing interest from academia and industry in enzymatic recycling of PA (polyamide) and PUR (polyurethane).^[^ ^6^, ^7^, ^8^, ^9^, ^10^, ^11^ ^]^ To this date, PA recycling processes are limited by the availability of depolymerases with sufficient performance. To realize a circular polyamide economy, sustained efforts in protein engineering of known depolymerases and the discovery of novel biocatalysts are indispensable.^[^ ^11^, ^12^, ^13^, ^14^ ^]^

As of now, the nylon hydrolase NylC_p2_ and its isoforms stand out as the only hydrolases to considerably depolymerize PA 6 and PA 6,6. NylC_p2_, originally isolated from *Arthrobacter* sp. KI72, has already undergone rational engineering to enhance the thermal stability, raising the melting temperature (*T*_m_) from 52 to 88 °C through the introduction of four substitutions. This resulted in the remarkably stable polyamidase NylC_p2_–TS (NylC_p2_^D36A/D122G/H130Y/E263Q^).^[^ ^15^, ^16^, ^17^ ^]^ Despite successfully enhancing the thermal stability, this achievement was accompanied by significant decrease in the turnover frequency.^[^ ^16^ ^]^ Consequently, the next step to advance enzymatic PA recycling is to design a polyamidase with activity and degree of depolymerization comparable to PETases used at industrial‐scale PET recycling (963 μmol_TPAeq_ h^−1^ mg_enzyme_^−1^; 98% in 24 h).^[^ ^1^ ^]^ In a recent study, we developed and validated a high‐throughput amine screening system for directed polyamidase evolution (AMIDE), which enables the engineering of polyamidases using directed evolution strategies that rely on high‐throughput screening, such as KnowVolution.^[^ ^12^ ^]^

KnowVolution is a versatile protein engineering strategy that has been used extensively to improve enzyme traits such as activity; thermal, ionic liquid, and pH resistance; substrate affinity; and substrate specificity.^[^ ^18^, ^19^, ^20^, ^21^, ^22^, ^23^ ^]^ KnowVolution combines computational analyzes with directed evolution to optimize properties with minimized experimental efforts and maximized improvements. The KnowVolution strategy comprises four phases: 1) identification of beneficial amino acid positions by screening random mutagenesis or smart libraries, 2) determination of beneficial substitutions for previously identified positions, 3) selection of positions to recombine concerning their epistasis, and 4) recombination of beneficial substitutions to maximize enzyme fitness.

Substrate docking is commonly applied for determining and evaluating the pose of a ligand (e.g., substrate) in complex with a receptor (e.g., enzyme) for applications such as virtual drug screening.^[^ ^24^ ^]^ Since docking is optimized for predicting the bound conformation and free energy of small molecules to the receptor, incremental approaches are necessary to extend its applicability to large molecules with many rotatable bonds, such as peptides.^[^ ^25^ ^]^ Recently, docking and subsequent molecular dynamics (MD) simulations of a small polymer model substrate (2PET) and a polymer‐degrading enzyme (PETase) were applied to decipher the effects of substitutions on enzyme activity at the molecular level.^[^ ^26^ ^]^ Aliphatic polycondensates (e.g., PA 6, PA 6,6, and PLA) display a significantly higher number of rotatable bonds than PET. Consequently, to enable the investigation of interactions between NylC_p2_–TS and PA 6, implementing an incremental docking workflow using oligomeric PA 6 substrates was deemed necessary. This approach would then allow reliable MD simulations to model the dynamic behavior of ligand‐receptor complexes.^[^ ^27^ ^]^

In this study, we present the first directed evolution campaign for a polyamidase and employed the KnowVolution strategy. We performed random mutagenesis and MD simulations of the enzyme‐substrate complex to identify key positions crucial for the interaction of NylC_p2_–TS with PA 6 and PA 6,6, and its hydrolysis. Recombination of the identified beneficial substitutions enabled us to enhance the specific activity of NylC_p2_–TS by up to 6.9‐fold. The finally obtained NylC–HP variant is the highest‐performing polyamidase to date in respect to PA 6 and PA 6,6 depolymerization efficiency. Our study highlights the potential of enzyme engineering to enhance enzymatic polyamide hydrolysis while also identifying limitations that need to be addressed in future efforts. Specifically, for NylC–HP, we propose that significantly widening the substrate binding pocket is necessary to achieve higher degrees of depolymerization.

## 2 Results and Discussion

The goal of this study is to generate knowledge to enable efficient engineering of polyamidases for PA 6 and PA 6,6 hydrolysis. We focused on enhancing the PA 6 depolymerization efficiency of NylC_p2_–TS and understanding the enzyme‐polymer interactions within the binding pocket that govern substrate hydrolysis. These aspects are described in three sections. First, KnowVolution is applied to engineer NylC_p2_–TS toward enhanced specific activity. Next, we experimentally characterize the highest‐performing variant (NylC–HP). Finally, we conduct computational analyzes to identify changes in enzyme‐enzyme and enzyme–substrate interactions that lead to improved substrate hydrolysis and provide molecular understanding of structural limitations for PA degradation.

Enzymatic PA recycling is an important research area for establishing a circular economy for synthetic polyamides and is gaining attention from academic research groups and industry.^[^ ^9^, ^10^, ^11^, ^28^ ^]^ Using characterized polymers as substrates and applying defined pretreatment techniques is essential for ensuring comparability between studies conducted by different research groups, as demonstrated in enzymatic PET degradation research.^[^ ^1^ ^]^ We observed background product formation in PA 6 and PA 6,6 degradation studies, primarily due to the extraction of unpolymerized monomers and the spontaneous hydrolysis of 6‐aminohexanoic acid (6–AHA) in the case of PA 6. In this study, all results were background‐corrected to monitor enzyme net activity. We aimed to maximize the reproducibility and comparability of this study. Thus, we utilized untreated Gf‐PA 6 film (Goodfellow GmbH, 0.2 mm thickness; *X*_c,PA 6_ = 21%; *M*_w,PA 6_ = 108.5 kDa; *M*_n,PA 6_ = 44.52 kDa) and Gf‐PA 6,6 granules (Goodfellow GmbH, 3 mm diameter; *X*_c,PA 6,6_ = 39%; *M*_w,PA 6,6_ = 61.84 kDa; *M*_n,PA 6,6_ = 29.30 kDa) and characterized them in respect to their crystallinity and molecular weight (Figure S1 and Table S1‐S2, Supporting Information). Recrystallization of PET during the enzymatic hydrolysis process significantly reduces depolymerization efficiency.^[^ ^1^, ^4^, ^10^, ^29^ ^]^ While the impact of PA crystallinity on enzymatic hydrolysis is not fully understood, it may also play a crucial role in enzymatic PA hydrolysis. Thus, we also determined the extent of PA 6 and PA 6,6 recrystallization during heat treatment mimicking enzymatic depolymerization process conditions. In a previous study, slight increases in crystallinity of PA 6 were observed when incubated at 60 and 70 °C for up to 10 days.^[^ ^10^ ^]^ From an economic perspective, it is unlikely to extend the process to several days.^[^ ^30^, ^31^ ^]^ To determine maximum process temperatures, we analyzed the crystallinity of Gf‐PA 6 film and Gf‐PA 6,6 granules after incubation at temperatures ranging from 40–90 °C for 24 h and confirmed that no significant increases in crystallinity occurred, even at 90 °C (Figure S1A,B and Table S1, Supporting Information). These findings suggest that, unlike enzymatic PET hydrolysis, both PA 6 and PA 6,6 depolymerization are not limited by recrystallization above the glass transition temperature (*T*_g, PA 6_ = 53 °C, *T*_g, PA 6,6_ = 57 °C).^[^ ^32^ ^]^ Therefore, PA depolymerization can be conducted at temperatures up to at least 90 °C, provided that appropriately thermostable enzymes are available.

### 2.1 MD Simulation‐Guided Directed Evolution of NylC_p2_–TS

We sought to increase the depolymerization efficiency of NylC_p2_–TS toward PA 6 by combining random mutagenesis and rational design (i.e., active site engineering performing docking and MD simulations) as part of a four‐step KnowVolution campaign. The specific activity of depolymerases is an established performance parameter and was used for evaluating and selecting NylC_p2_–TS variants.^[^ ^2^, ^4^, ^33^ ^]^

#### 2.1.1 Identification of Potentially Beneficial Positions Through Random Mutagenesis and MD Simulations

**Figure 1:**
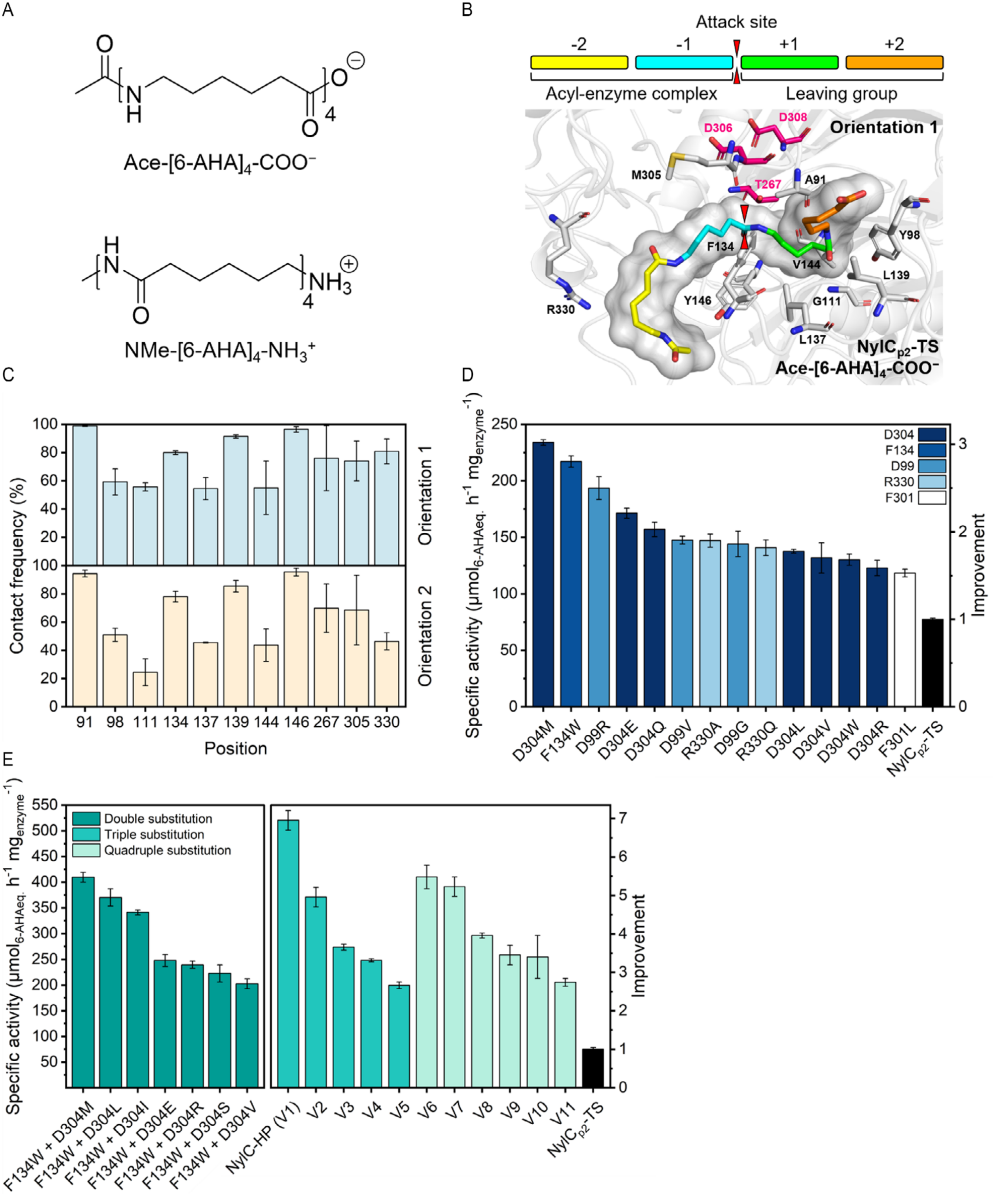
MD simulation‐guided directed evolution of NylC_p2_–TS. A) PA 6 tetramers used for substrate docking and MD simulation. Ace–[6–AHA]_4_–COO^−^ was used as proxy for the PA 6 polymer chain carboxy‐terminus and NMe–[6–AHA]_4_–NH_3_^+^ for the amine‐terminus. The opposing termini were capped to mimic the continuation of the polymer chain. B) Representative structure of enzyme‐substrate complex NylC_p2_–TS/Ace–[6–AHA]_4_–COO^−^(orientation 1). Residues with ≥50% of the simulation time in ≤2.5 Å contact sphere of the enzyme are shown as sticks. The catalytic triad is highlighted in pink. C) Contact frequencies of the ≤2.5 Å contact sphere of NylC_p2_–TS and both Ace–[6–AHA]_4_–COO^−^ orientations. D) Specific activities of NylC_p2_–TS single substitutions. Specific activities were calculated from released 6–AHA equivalents after incubation with Gf‐PA 6 film (50 nM/1.90 mg L^−1^ enzyme, 456 g L^−1^ Gf‐PA 6, 50 mM bicine, pH 8.0, 100 mM NaCl, 60 °C, 2 h). E) Specific activities of NylC_p2_–TS double‐, triple‐, and quadruple substitutions. Specific activities of double substitutions were calculated from released 6–AHA equivalents after incubation with Gf‐PA 6 film (25 nM/0.95 mg L^−1^ enzyme, 456 g L^−1^ Gf‐PA 6, 50 mM bicine, pH 8.0, 100 mM NaCl, 60 °C, 2 h). Specific activities of triple‐ and quadruple substitutions were calculated from the *V*max obtained from conventional Michaelis‐Menten kinetics (25 nM/0.95 mg L^−1^ enzyme, 76–608 g L^−1^ Gf‐PA 6, 50 mM bicine, pH 8.0, 100 mM NaCl, 60 °C, 2 h). Reactions were conducted in at least duplicate. Error bars represent the standard error of the mean.

The first step of the KnowVolution strategy comprises the identification of potentially beneficial positions. We applied random mutagenesis to identify beneficial positions across the entire gene and MD simulations of the enzyme‐substrate complex to determine positions involved in enzyme–substrate interactions. From screening a random mutagenesis library comprising 1700 clones for specific activity toward Gf‐PA 6 via the AMIDE method, we identified eleven potentially beneficial positions (V18, L24, P27, F38, D99, F100, G111, A160, D209, F301, and D304). For in silico identification, we determined the contact frequency of the individual residues of the enzyme with the substrate observed in the MD simulations. To generate the enzyme‐substrate complexes, the PA 6 tetramers Ace–[6–AHA]_4_–COO^−^ and NMe–[6–AHA]_4_–NH_3_^+^; comprising four repeating units of 6–AHA, one capped end representing the continuation of the polymer chain, and one charged end representing the terminus of the polymer chain; were incrementally docked into the binding pocket of NylC_p2_–TS (**Figure** **1**A and B). We observed two substrate orientations with the tetramer's attack site near the active site but with mirrored substrate termini (Figure S2, Supporting Information). NylC_p2_–TS exhibited a lower free energy of binding for Ace–[6–AHA]_4_–COO^−^ in both orientations (orientation 1: Ace–[6–AHA]_4_–COO^−^ and orientation 2: ^−^OOC–[6–AHA]_4_–Ace) compared to NMe–[6–AHA]_4_–NH_3_^+^ (Figure S2 and Table S3, Supporting Information).

Therefore, MD simulations of the enzyme‐substrate systems NylC_p2_–TS/Ace–[6–AHA]_4_–COO^−^ (orientation 1) and NylC_p2_–TS/^−^OOC–[6–AHA]_4_–Ace (orientation 2) were performed. We considered a residue to be in contact with the substrate when the distance between any atom of the two was less than or equal to 2.5 Å. Figure 1B displays a representative structure of the NylC_p2_–TS/Ace–[6–AHA]_4_–COO^−^ enzyme‐substrate complex, with interacting residues highlighted. The reported contact frequencies represent the mean of three independent 150 ns simulation runs and give the percentage of simulation time of existing contacts (Figure 1C). Positions that were in contact with the substrate for at least 50 % of the simulation time were selected for experimental testing. The MD simulations revealed ten positions (A91, Y98, G111, F134, L137, L139, V144, Y146, T267, M305, and R330) to be in the 2.5 Å interaction sphere for at least 50 % of the simulation time (Figure 1B and C). Even though T267 showed an average contact frequency of ≥50%, it was not considered for site‐saturation, as it acts as nucleophilic residue in the catalytic triad and is deemed indispensable for substrate hydrolysis.^[^ ^16^, ^34^ ^]^

#### 2.1.2 Determining Beneficial Substitutions Through Site‐Saturation Mutagenesis

**Figure 2:**
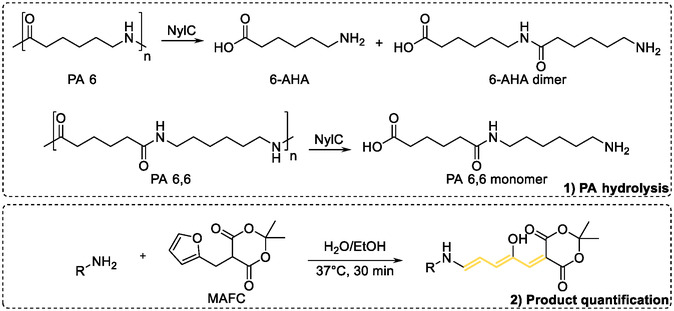
Enzymatic conversion of PA 6 and PA 6,6 and quantification of released amines. NylC‐type enzymes hydrolyze PA 6 into 6‐AHA and its dimer. PA 6,6 is hydrolyzed into its monomeric unit, consisting of hexamethylene diamine and adipic acid. The released amines are subsequently labeled with MAFC (Meldrum's acid furfural conjugate) and quantified using a photospectrometer.

In the second step, site‐saturation mutagenesis libraries for 20 positions (i.e., V18, L24, P27, F38, A91, Y98, D99, F100, G111, F134, L137, L139, V144, Y146, A160, D209, F301, D304, M305, and R330) were generated using degenerate primers (NNK) and screened with Gf‐PA 6 film as substrate via the AMIDE method to identify substitutions leading to improved specific activity.^[^ ^12^ ^]^ NylC‐type enzymes hydrolyzes PA 6 primarily into 6‐AHA and the 6‐AHA dimer, and PA 6,6 into its monomeric unit, comprising hexamethylene diamine and adipic acid (**Figure** **2**). We quantified 6–AHA equivalents by labeling released amines, serving as indicators of hydrolysis reactions catalyzed by the enzyme. The specific activity is expressed as μmol_6–AHAeq_. h^−1^ mg_enzyme_^−1^. Out of 20 positions, substitutions at five positions (D99, F134, F301, D304, and R330) showed significantly improved activity (1.5‐ to 3.0‐fold) compared to NylC_p2_–TS (Figure 1D). Notably, all of these positions are located near the substrate‐binding pocket, either surface‐exposed within the pocket itself (D99, F134, D304, R330) or buried just behind the active site threonine (F301). For D99(R/G/V), F134W, F301L, and R330(A/Q) a small set of 1‐3 amino acid substitutions showed improved activity. Interestingly, position 304 exhibited significant variability, as many substitutions D304(M/E/Q/L/V/R/W), resulted in enhanced PA 6 hydrolysis activity.

#### 2.1.3 Selection of Positions for Recombination

In the third step, we selected positions for which we determined beneficial substitutions for recombination. The recombination of beneficial substitutions can lead to negative epistasis, resulting in only moderate improvements or even a reduction in fitness, when individually advantageous substitutions do not exhibit additive effects.^[^ ^35^ ^]^ Epistasis of recombined amino acid substitutions is more likely to occur when positions are in close spatial proximity, as in the case of F301 and D304.^[^ ^36^ ^]^ To assess epistasis, we evaluated the compatibility of substitutions at these positions. We screened an iterative site‐saturation mutagenesis library where D304 was saturated using NylC_p2_–TS^F301L^ as the parental enzyme. This resulted in negative epistasis for all F301L/D304X double‐substituted variants. Due to negative epistasis and the fact that F301L displayed only a weak beneficial effect on the activity of NylC_p2_–TS (1.5‐fold ± 0.1 improved specific activity), we excluded F301L and continued recombination with beneficial substitutions at the remaining positions (i.e., D99, F134, D304, and R330).

#### 2.1.4 Recombination of Determined Beneficial Substitutions Yields a Polyamidase with High Performance

In the fourth and final step, we recombined beneficial positions to maximize the activity of NylC_p2_–TS. The number of beneficial substitutions at each position differed greatly, and most prominently, seven substitutions at position D304 increased the specific activity of NylC_p2_–TS significantly (1.6 to 3.0‐fold) (Figure 1D). The variant with the substitution F134W exhibited the second‐highest catalytic improvement, with a single beneficial phenylalanine‐to‐tryptophan substitution increasing the specific activity (2.8‐fold ± 0.1). Substitutions at D99(R/G/V) and R330(A/Q) enhanced the specific activity, with three substitutions at position D99 (1.9‐ to 2.5‐fold) and two at position R330 (1.8‐ to 1.9‐fold). The best single substitution, D304M resulted in a 3.0‐fold ± 0.1 improved activity. Introducing methionine at position D304 may, however, not necessarily show the highest synergy when recombined with F134W. Hence, iterative site‐saturation is advisable, wherein variants with improved activity are used as a new reference point for another round of screening.^[^ ^37^, ^38^ ^]^ Accordingly, we site‐saturated D304 with NylC_p2_–TS^F134W^ as the parental enzyme to trace the NylC_p2_–TS^F134W/D304X^ double‐substituted variant with the highest specific activity. 45 clones exhibiting higher activity than the parental enzyme were sequenced. Among them, NylC_p2_–TS^F134W/D304M^ was the most frequently occurring variant (13%) and showed the most improved specific activity for PA 6 (5.3–fold ± 0.1) (Figure 1E, Figure S3, and Table S4–S5, Supporting Information). Following this double substitution, eleven recombined variants with D99(R/G/V) and R330(A/Q) remained for evaluation. We generated all possible triple and quadruple substitutions based on NylC_p2_–TS^F134W/D304M^ (V1–V11, Table S5, Supporting Information) and determined respective specific activities (Figure 1E and Figure S4, Supporting Information). The triple‐substituted variant NylC_p2_–TS^F134W/D304M/R330A^ (NylC–HP) achieved the highest performance, exhibiting a 6.9‐fold ± 0.3 improved specific activity for PA 6. Introducing a fourth substitution at position 99 (V6–V8), particularly D99R (V8), resulted in impaired activity (Table S5, Supporting Information). What molecular interactions at this position affect polyamidase activity will be further evaluated in the computational analysis section of this study. Finally, the specific activity of NylC–HP could be enhanced from 77 ± 1.0 to 520 ± 19 μmol_6–AHAeq_ h^−1^ mg_enzyme_^−1^ in the first directed polyamidase evolution campaign. This brings the specific activity of NylC–HP within the range of the most efficient PETases (e.g., 963 μmol_TPAeq_ h^−1^ mg_enzyme_^−1^ for LCC^ICCG^), making NylC–HP the fastest polyamidase reported to this date.^[^ ^1^ ^]^

### 2.2 Experimental Characterization of NylC–HP

**Figure 3:**
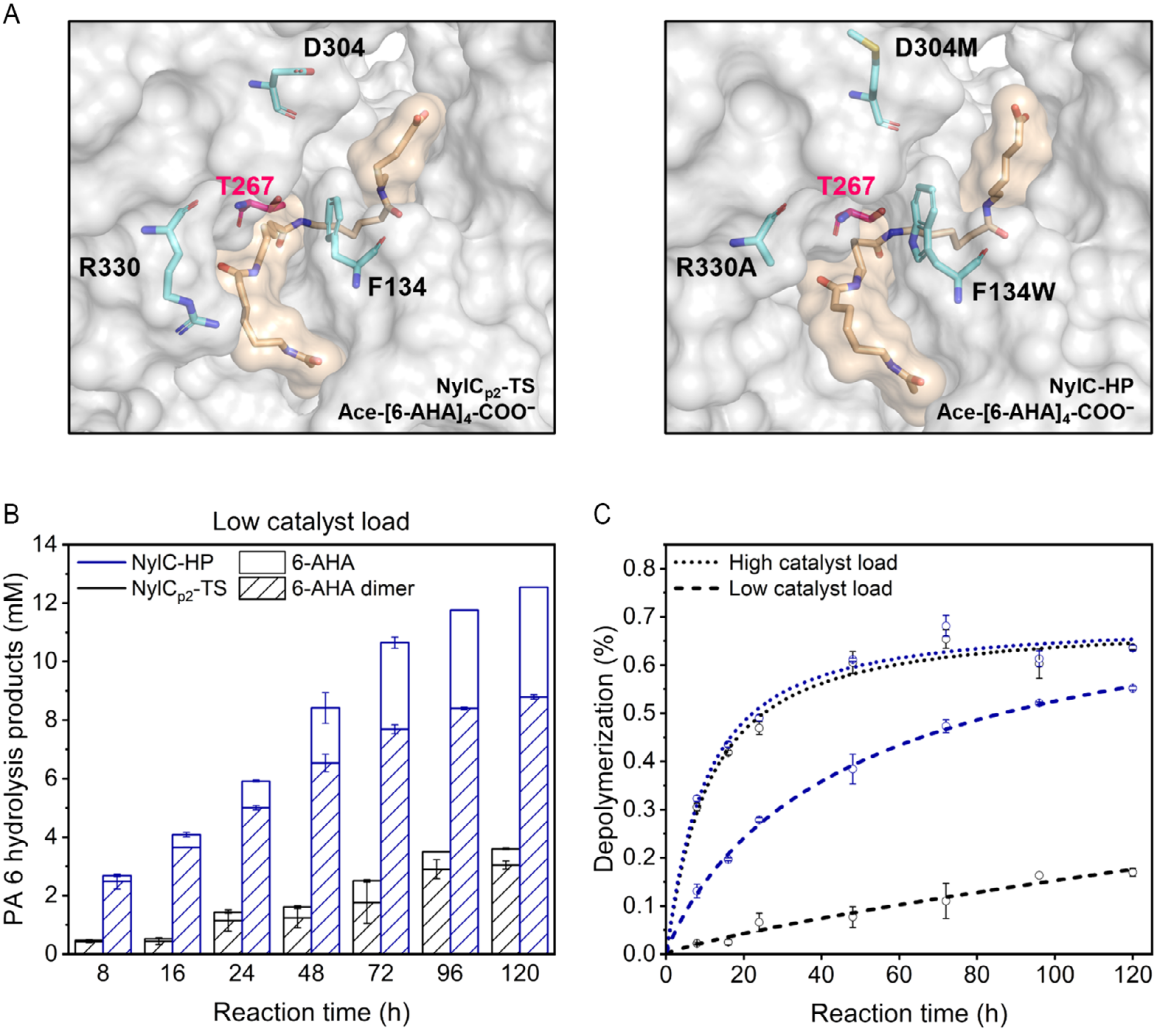
Experimental characterization of NylC–HP. A) Representative structure of the enzyme‐substrate complexes NylC_p2_–TS/Ace–[6–AHA]_4_–COO^−^ and NylC–HP/Ace–[6–AHA]_4_–COO^−^(orientation 1). Introduced amino acid substitutions are shown as turquoise sticks. The catalytic threonine T267 is highlighted in pink. B) Time‐resolved Gf‐PA 6 degradation by NylC_p2_–TS and NylC–HP with low catalyst load (50 nM/1.90 mg L^−1^ enzyme, 456 g L^−1^ Gf‐PA 6, 50 mM bicine, pH 8.0, 100 mM NaCl, 60 °C, 8–120 h). Blue bars represent NylC–HP, and black bars represent NylC_p2_–TS. Dashed bars indicate the 6‐AHA dimer, while solid (blank) bars indicate 6‐AHA. C) Degree of Gf‐PA 6 depolymerization by NylC_p2_–TS and NylC–HP at low and high catalyst load. Blue lines represent NylC–HP, and black lines represent NylC_p2_–TS. Reactions were conducted in at least duplicates. Error bars represent the standard error of the mean.

We optimized NylC_p2_–TS through directed evolution to enhance the PA 6 degradation efficiency and obtained a polyamidase with high performance harboring three substitutions (NylC_p2_–TS^F134W/D304M/R330A^; NylC–HP) (**Figure** **3**A). In the following, we characterized NylC–HP experimentally.

#### 2.2.1 NylC–HP Preferentially Degrades PA 6,6 over PA 6

We selected NylC_p2_–TS variants based on their PA 6 depolymerization efficiency with an up to 6.9–fold increased specific activity (Figure 1E and Figure S4, Supporting Information). We also assessed the specific activity of NylC‐HP toward PA 6,6 by detecting the release of the PA 6,6 monomer (6‐[(6‐Aminohexyl)amino]‐6‐oxo‐hexanoic acid), the primary product of enzymatic PA 6,6 hydrolysis.^[^ ^28^ ^]^ Consistent with the results for PA 6, we observed a 3.4–fold ± 0.1 increase in specific activity toward PA 6,6 for NylC–HP (i.e., from 304 ± 9 μmol_PA6,6monomer_ eq h^−1^ mg_enzyme_^−1^ to 1026 ± 25 μmol_PA6,6monomer_ eq h^−1^ mg_enzyme_^−1^) compared to NylC_p2_–TS (Figure S5, Supporting Information). Given that PA 6 hydrolysis was the primary selection pressure in our campaign, we anticipated the most significant improvement in specific activity toward PA 6. Interestingly, even though the achieved relative improvement of NylC–HP is higher for PA 6 hydrolysis (6.9‐fold ± 0.3) compared to PA 6,6 (3.4‐fold ± 0.1), the enzyme still hydrolyzes PA 6,6 two times faster than PA 6. This result is even more striking considering that NylC–HP does not fully hydrolyze PA 6,6 to hexamethylene diamine and adipic acid but stops at the PA 6,6 monomer (Figure S6, Supporting Information). In contrast, PA 6 is at least partially converted to 6–AHA (Figure 3B).^[^ ^28^ ^]^ These observations emphasize that PA 6 and PA 6,6, despite sharing similar chemical and physical properties, undergo distinct enzymatic processing. A similar behavior was reported for the reaction kinetics of *Is*PETase for the semi‐aromatic polymers PET and PEF, where the enzyme favored the furan‐based PEF over the benzene‐based PET.^[^ ^39^ ^]^

#### 2.2.2 NylC–HP Depolymerizes PA 6 Efficiently but Limited Degree of Depolymerization

Parameters for the economic assessment of enzymatic polymer recycling processes have been recently defined as substrate loading, conversion/degree of depolymerization, and reaction kinetics, which together entail enzyme productivity.^[^ ^1^, ^3^ ^]^ To maximize space‐time yields, substrate loading is best optimized by process engineering. For instance, polymers can be pretreated to maximize substrate loading and surface of exchange and minimize polymer crystallinity.^[^ ^1^, ^30^, ^40^ ^]^ In contrast, reaction kinetics and degree of depolymerization represent promising targets for protein engineers.^[^ ^2^, ^41^, ^42^ ^]^ Established metrics for assessing the performance of heterogeneous biocatalysts include monitoring the maximum reaction rate under substrate saturation/inverse Michaelis–Menten conditions (^inv^MM), which reveals the number of attack sites the depolymerase can effectively target, and under enzyme saturation/conventional Michaelis‐Menten conditions (^conv^MM), which provides a measure of the maximum reaction rate of the depolymerization reaction.^[^ ^43^ ^]^ The degree of depolymerization can be displayed by ‘the ultimate mass’ fraction of depolymerized product but must be carefully distinguished from factors such as improved reaction kinetics, which can outperform polymer recrystallization and consequently increase the fraction of depolymerized polymer.^[^ ^9^ ^]^

To assess the kinetic efficiency of NylC–HP beyond initial rates, we conducted long‐term degradation experiments approaching enzyme saturation (low catalyst load; c_enzyme_ = 50 nM, c_PA 6_ = 456 g L^−1^) and substrate saturation (high catalyst load; c_enzyme_ = 5 μM, c_PA 6_ = 456 g L^−1^), determined the respective product composition, and subsequently computed the corresponding degree of depolymerization. At low catalyst load, NylC–HP exhibited a 5.8‐fold ± 0.7 enhanced initial specific activity of 39.4 ± 4.2 mg_6–AHAeq_. h^−1^ mg_enzyme_^−1^ within the first 8 h of the reaction, compared to 6.8 ± 0.6 mg_6–AHAeq_. h^−1^ mg_enzyme_^−1^ for NylC_p2_–TS (Figure 3B). The average specific activity was 3.3‐fold ± 0.1 higher with 11.1 ± 0.1 mg_6–AHAeq_. h^−1^ mg_enzyme_^−1^ for NylC–HP and 3.4 ± 0.2 mg_6–AHAeq_. h^−1^ mg_enzyme_^−1^ for NylC_p2_–TS throughout 120 h of reaction. At high catalyst load, NylC–HP and NylC_p2_–TS exhibited comparable initial and average specific activities of 97.2 ± 1.9 and 91.8 ± 2.4, and 12.7 ± 0.1 and 12.8 ± 0.1 mg_6–AHAeq_. h^−1^ mg_enzyme_^−1^, respectively (Figure S7, Supporting Information). The latter observation coincides with the final degree of depolymerization, where both enzymes stalled around 0.64 ± 0.01% (w/w) (Figure 3C). This highlights that increasing the number of targetable attack sites and the degree of depolymerization is crucial to unlock the potential of polyamidases for PA recycling.

#### 2.2.3 NylC–HP Shows Higher Conversion to the PA 6 Monomer than NylC_p2_–TS

The enzymatic depolymerization of PA 6 is complex, involving the hydrolysis of distinct attack sites on the polymer surface and oligomers with different chain lengths. This makes determining reaction rates for individual partial reactions challenging. The two main products of PA 6 hydrolysis are 6–AHA and the corresponding dimer. Tuning the conversion toward 6–AHA is desirable since, in a circular process, 6–AHA can be converted to ε‐caprolactam and repolymerized to virgin‐grade PA 6.^[^ ^44^ ^]^ After 120 h, NylC–HP achieved notably higher rates of complete depolymerization compared to the parental enzyme. Specifically, 30% (low catalyst load) and 90% (high catalyst load) of the released products were fully depolymerized to 6–AHA, whereas the parental enzyme achieved only 15% and 80%, respectively (Figure 3B and Figure S7, Supporting Information).

#### 2.2.4 NylC–HP Possesses Enhanced Thermal Stability

**Figure 4:**
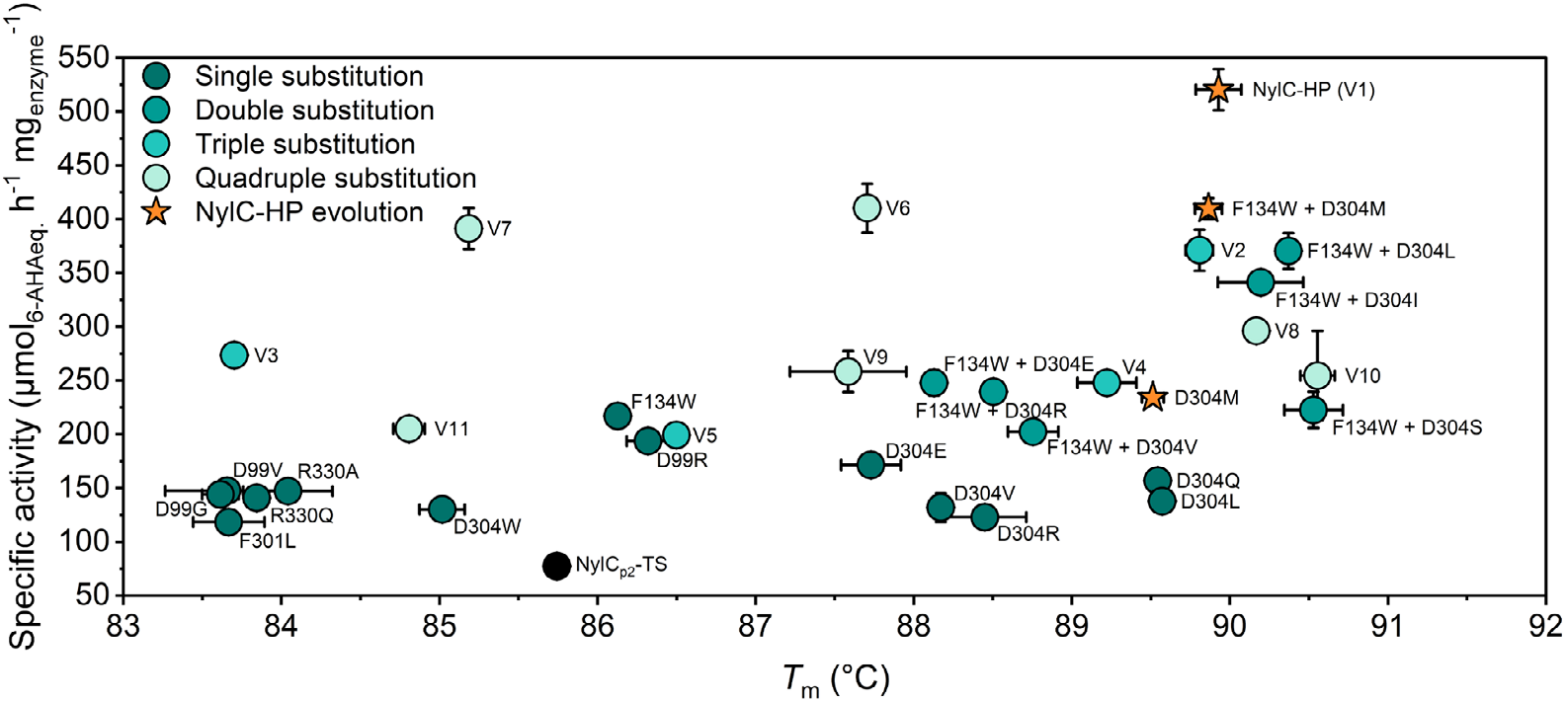
Thermal stability and specific activity of NylC_p2_–TS variants. Melting temperatures (*T*_m_) of NylC_p2_–TS variants are plotted against their specific activity. The parental enzyme, NylC_p2_–TS, is shown in black, with variants of increasing degrees of substitution represented in progressively brighter turquoise tones, and the evolutionary trajectory of NylC–HP highlighted in orange. Reactions were conducted in at least duplicates. Error bars represent the standard error of the mean.

Next to depolymerase activity, thermal stability plays a pivotal role in enzymatic polymer recycling, enabling accelerated reaction rates at elevated temperatures and reducing catalyst deactivation during the process.^[^ ^45^, ^46^ ^]^ Consequently, we determined melting temperatures for all tested variants (**Figure** **4**). Substitutions at positions D99, F301, and R330 generally reduced thermal stability, except for a slight increase for D99R. The substitution F134W had no impact on thermal stability. Interestingly, all single substitutions at D304 except D304W increased thermal stability by up to 3.8 ± 0.1 °C and for all NylC_p2_–TS^F134W/D304X^ double substitutions by up to 4.8 ± 0.2 °C. Although NylC_p2_–TS is already thermostable (*T*_m_ = 85.7 ± 0.1 °C), we were able to further improve the thermal stability of NylC–HP by an additional 4.2 ± 0.2 °C, achieving a remarkable *T*_m_ of 89.9 ± 0.1 °C. The enhanced specific activity underlines NylC–HP's PA 6 depolymerization efficiency, leading to enhanced conversion rates and an increased monomer yield. The degree of depolymerization, however, remains a limiting factor, as previously reported.^[^ ^10^, ^12^ ^]^ Regardless, the development of NylC–HP represents hitherto unmatched enzymatic PA 6 and PA 6,6 depolymerization efficiency together with excellent thermal stability, rendering NylC–HP not only the fastest but also one of the most thermostable characterized polyamidases to this date.

### 2.3 Elucidating Structure‐Function Relationships Using Docking and MD Simulations

All positions that led to increased specific activity are located either around the active site or directly within the substrate‐binding pocket. While this is to be expected for positions identified through analysis of enzyme–substrate interactions, it also applies to positions F301 and D304, which were not identified through structural analysis but rather emerged from random mutagenesis. Accordingly, it would not be surprising if substitutions at these positions that lead to improved specific activity also affected substrate interaction. To gain insights into molecular changes of enzyme–substrate interactions induced by the most beneficial substitutions D99R, F134W, D304M, and R330A, we generated enzyme‐substrate complexes, using NylC_p2_–TS, NylC–HP, and NylC–HP^D99R^ as enzymes and Ace–[6–AHA]_4_–COO^−^ and NMe–[6–AHA]_4_–NH_3_^+^ as substrates. We applied an incremental docking procedure (Figure S8–S9, Supporting Information), followed by MD simulations (Figure S10–S12, Supporting Information), and compared the dynamic behavior of examined enzyme‐substrate complexes. Moreover, we studied the dynamics of systems containing–besides water and ions–only the enzyme (i.e., NylC_p2_–TS, NylC_p2_–TS^D99R^, NylC–HP, and NylC–HP^D99R^) to determine differences in protein–protein interactions (Figure S10–S13, Supporting Information). In addition to the identification of activity‐enhancing molecular changes, deciphering the driving force that causes the negative epistasis that was observed upon combination of D99R and D304M, and structural features limiting the degree of depolymerization was of particular interest.

#### 2.3.1 Improved Variants NylC–HP and NylC–HP^D99R^ Prefer Different Substrate Orientations than NylC_p2_–TS

The incremental docking procedure revealed differences in the substrate poses that were ranked best (high population and affinity) for each enzyme. While for NylC_p2_–TS both orientations of the substrate Ace–[6–AHA]_4_–COO^−^ led to the energetically most favored conformations, the best substrate poses for NylC–HP and NylC–HP^D99R^ could be achieved with Ace–[6–AHA]_4_–COO^−^ (orientation 1) and NMe–[6–AHA]_4_–NH_3_^+^ (orientation 2) (Figure S2, Table S3, and Table S7,S8, Supporting Information).

#### 2.3.2 D99 and R330 Stabilize Substrate Termini Through Electrostatic Interactions

**Figure 5:**
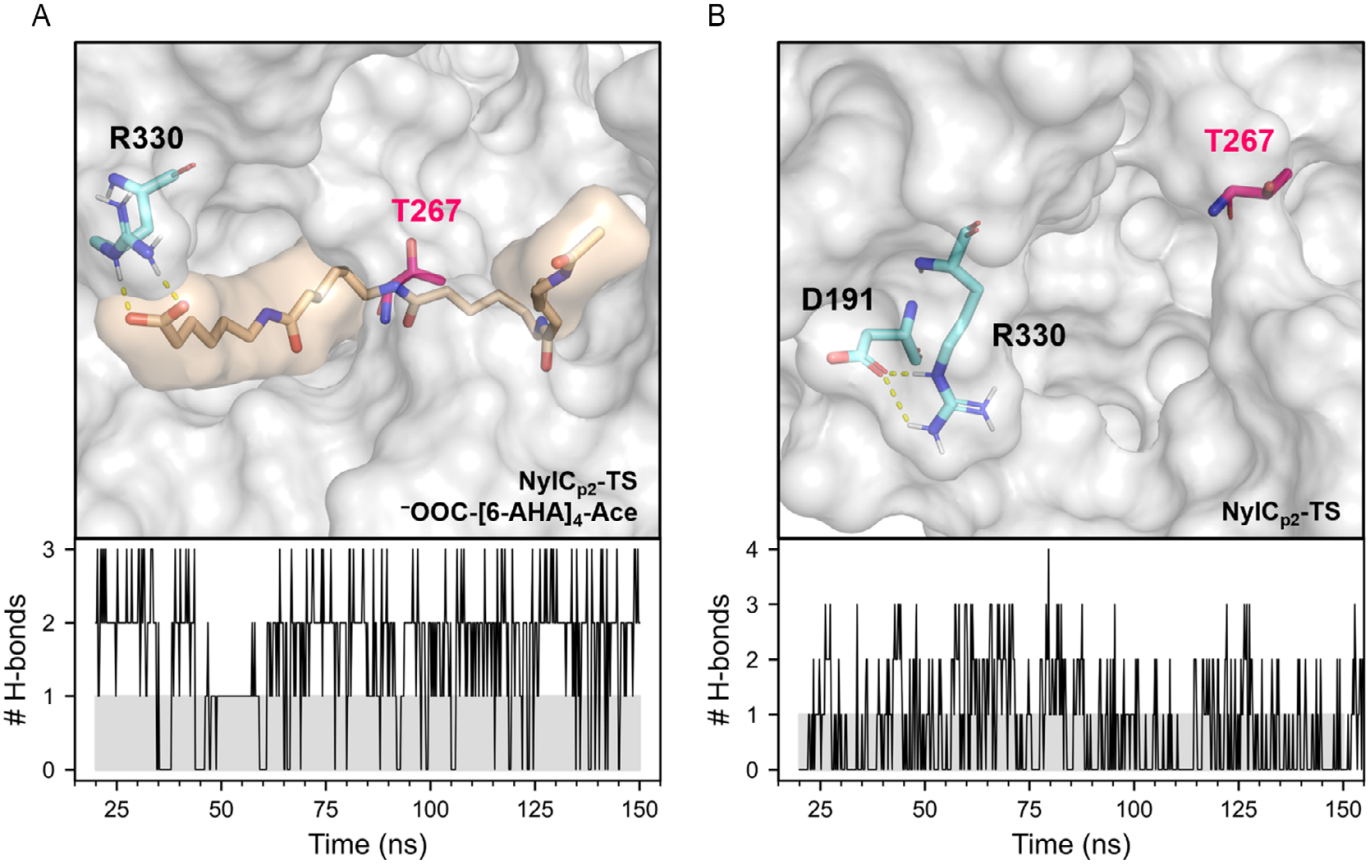
Hydrogen bonds (H—bonds) of residue R330 observed during MD (MD) simulations. A) Representative structure of the enzyme‐substrate complex NylC_p2_–TS/^−^OOC–[6–AHA]_4_–Ace with established H—bonds between the side chain of residue R330 and the negatively charged terminus of the substrate (top) and the number of such bonds (# H—bonds) in dependency of the simulation time observed for one MD simulation of this system (bottom). B) Representative structure of NylC_p2_–TS with established H—bonds between the side chains of residues R330 and D191 (top) and the number of such bonds (# H—bonds) in dependency of the simulation time observed for one MD simulation of this system (bottom).

**Figure 6:**
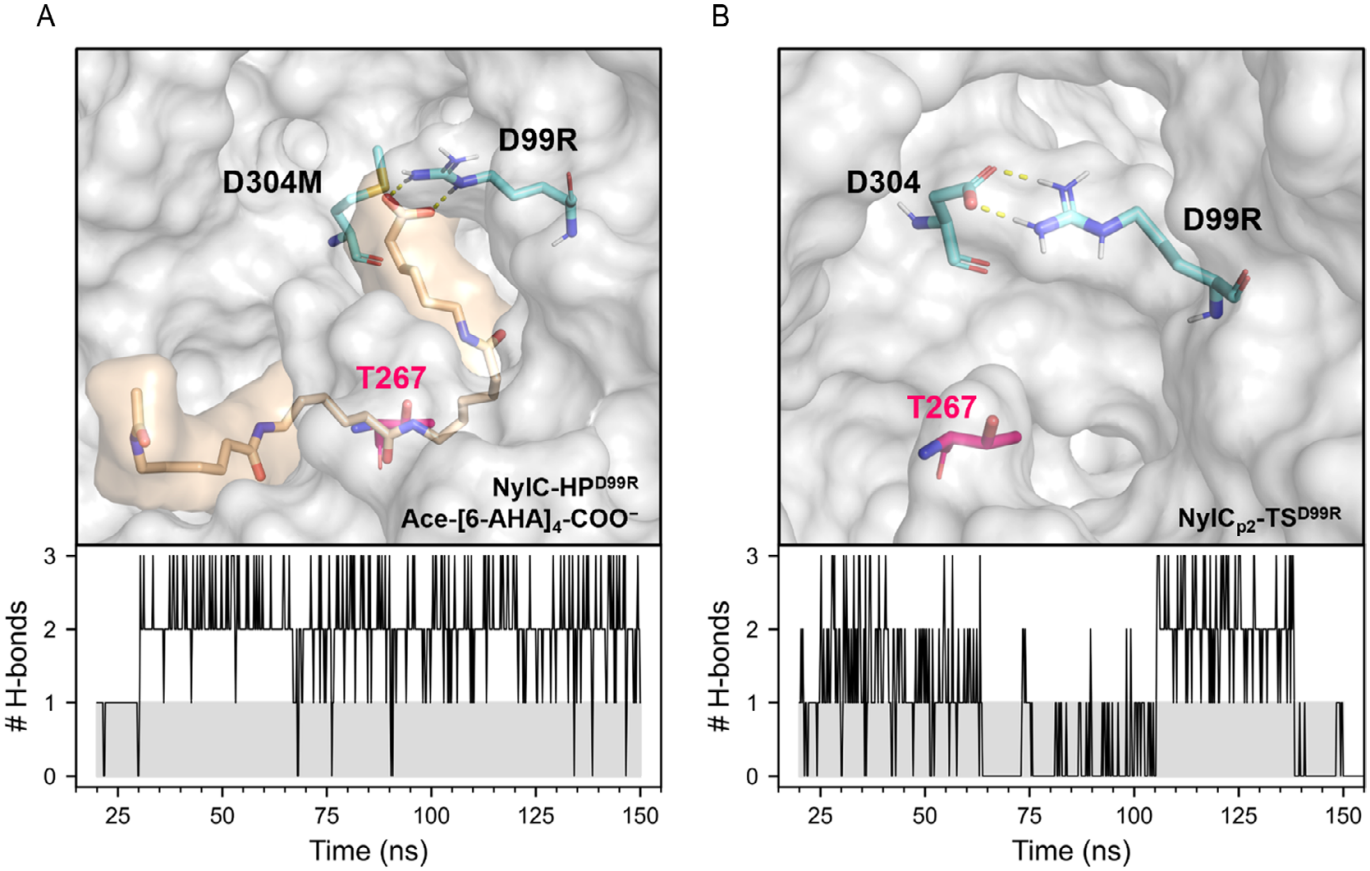
Hydrogen bonds (H—bonds) of residue D99R observed during MD (MD) simulations. A) Representative structure of the enzyme‐substrate complex NylC_p2_–TS^D99R^/Ace–[6–AHA]_4_–COO^−^ with established H—bonds between the side chain of residue D99R and the negatively charged terminus of the substrate (top) and the number of such bonds (# H—bonds) in dependency of the simulation time observed for one MD simulation of this system (bottom). B) Representative structure of NylC_p2_–TS^D99R^ with established H—bonds between the side chains of residues D99R and D304 (top) and the number of such bonds (# H—bonds) in dependency of the simulation time observed for one MD simulation of this system (bottom).

During simulation we observed that the residues at positions D99 and R330 interact with the charged termini of the polymer, COO^−^ and NH_3_^+^ (**Figure** **5**A and **6**A). At least one hydrogen bond (H‐bond) or salt bridge between the side chain of R330 and the negatively charged terminus of the substrate was formed for 67± 16% (H‐bond) and 65 ± 18% (salt bridge) of the simulation time, respectively (Figure 5A and Table S9, Supporting Information). In addition, we found a significant drop in interaction energy (sum of short‐range Lennard‐Jones and Coulomb interaction energy) between the enzyme and the substrate upon H‐bond formation with R330 Δ*E* = −98 ± 18 kJ mol^−1^ (Table S10, Supporting Information). Variants comprising the substitution R330A showed a significantly reduced contact frequency (*f*) between the residue at this position and the substrate compared to NylC_p2_–TS (Δ*f*(NylC–HP, NylC_p2_–TS = −51 ± 11% and Δ*f*(NylC–HP^D99R^, NylC_p2_–TS) = −47 ± 11%). Depolymerases tend to exhibit excessively strong binding to their polymer substrates, and reducing binding affinity has been shown to enhance activity.^[^ ^47^ ^]^ As observed during MD simulations, exchanging arginine with alanine reduces electrostatic interactions between enzyme and substrate, which may explain the increased specific activity observed upon introducing the R330A substitution.

In addition to altered enzyme–substrate interactions, we also observed differences in protein–protein interactions, which manifested itself in the dissolution of the salt bridge between the residues D191 and R330 (Figure 5B). While the formation of such a salt bridge could be observed up to 28% of the simulation time for the NylC_p2_–TS variant, it was absent for all variants carrying the substitution R330A (Table S11, Supporting Information). It has been shown that the (thermal) stability of a protein strongly correlates with the amount of noncovalent interactions present in the structure (e.g., H‐bonds, salt bridges, and hydrophobic interactions).^[^ ^48^, ^49^, ^50^ ^]^ We propose that the measured reduced thermal stability of NylC_p2_–TS^R330A^ compared to NylC_p2_–TS may be explained by the omission of the structure–stabilizing interaction D191‐R330 when introducing the substitution R330A.

The increase in specific activity of NylC–HP was achieved by substituting F134W, D304M, and R330A (Figure 1E and Table S5, Supporting Information). Introducing D99R to NylC_p2_–TS also increased enzyme activity, however, when introduced to NylC–HP, negative epistasis was observed. In particular, the improvement in specific activity decreased from 6.9‐ to 3.9‐fold for NylC–HP and NylC–HP^D99R^. For NylC–HP^D99R^, the MD simulations revealed persistent H‐bond formation (up to 98% of the simulation time) between D99R and the negatively charged terminus of the substrate Ace–[6–AHA]_4_–COO^−^ (Figure 6A and Table S9, Supporting Information). We determined the interaction energy between the enzyme and the substrate for this system and observed a significant drop of Δ*E* = −92 ± 26 kJ mol^−1^ upon H‐bond formation between D99R and the carboxy‐terminus of the substrate (Table S10, Supporting Information). For NylC_p2_–TS^D99R^, we observed the formation of a salt bridge between D99R and D304 during simulation (39 ± 10% of the simulation time) (Figure 6B and Table S11, Supporting Information). This salt bridge might also explain the increase in *T*_m_ observed for NylC_p2_–TS^D99R^ (Figure 4).

Based on the MD results described above, we assume that reducing interactions between residue 99 and the charged terminus of the polymer supports substrate hydrolysis. In the case of NylC_p2_–TS^D99R^, substrate interactions may be reduced, as salt bridge formation between the guanidino group of D99R and the carboxy group of D304 could prevent D99R from interacting with the substrate. However, when residue at position 304 cannot serve as a H‐bond acceptor, as in NylC–HP^D99R^, the interaction between D99R and the substrate may be restored, leading to a decrease in enzyme activity.

#### 2.3.3 Positions 304 and 305 Are Nonconserved Islands in a Highly Conserved Region

**Figure 7:**
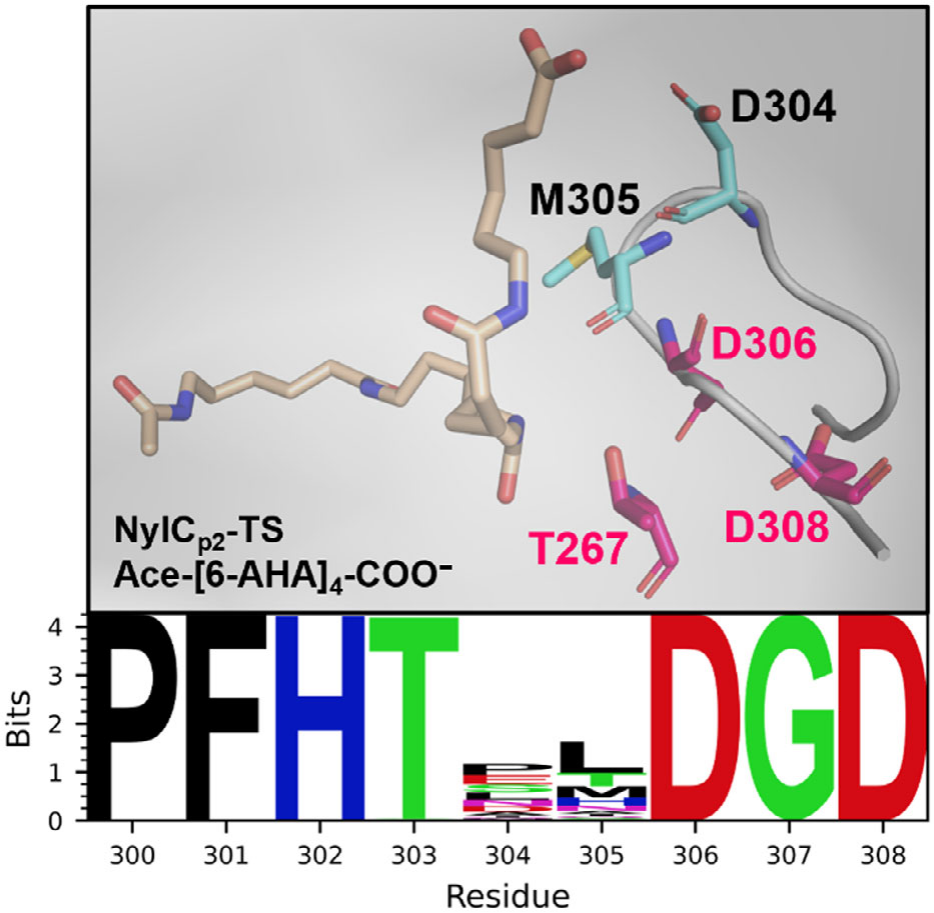
Residues D304 and M305 represent nonconserved amino acids within a highly conserved region of NylC_p2_–TS that includes two residues of the catalytic triad (i.e., D306 and D308). Detailed view of the highly conserved region including positions 300–308 of NylC_p2_–TS, with the nonconserved residues highlighted in blue and the residues of the catalytic triad highlighted in pink (top). Excerpt from the sequence logo of NylC_p2_–TS (bottom).

The strong beneficial impact on NylC_p2_–TS activity and thermal stability of all tested D304X substitutions suggests that the naturally occurring aspartate causes a structurally unfavorable conformation of the enzyme. Conservation analysis showed that positions D304 and M305 constitute the sole highly variable positions within a predominantly conserved patch spanning residues of the catalytic triad (i.e., D306 and D308) (**Figure** **7**). Interestingly, in contrast to saturating position D304, saturating position M305 led to no variant with notable polyamidase activity.

#### 2.3.4 Conformational Dynamics of Aromatic Sidechains at Position 134 Control Substrate Binding and Hydrolysis

**Figure 8:**
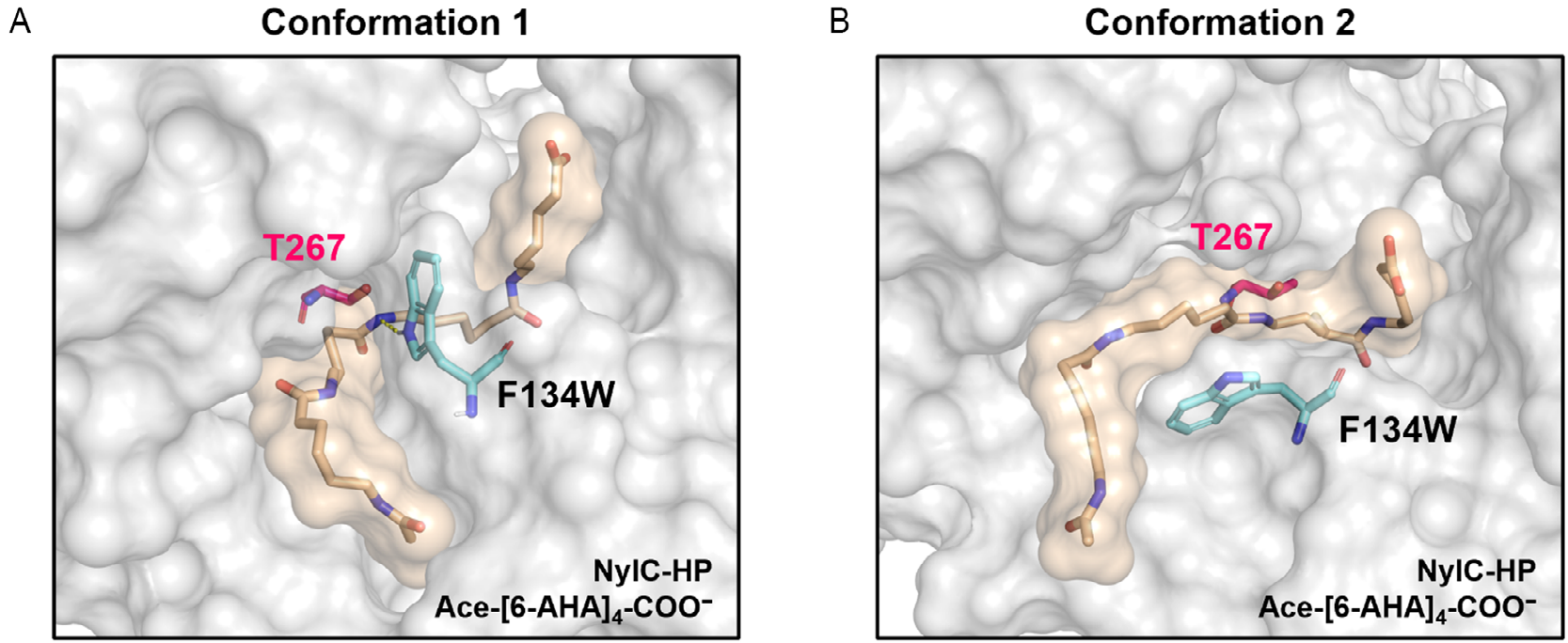
Representative structures highlighting the two main conformations of residue F134W observed during MD simulations of the enzyme‐substrate complex NylC–HP/Ace–[6–AHA]_4_–COO^−^. A) Conformation 1 is characterized by a vertically oriented side chain of F134W, which prevents intramolecular interactions and (optionally) fixes the amide bond by H—bonding close to the active residue T267. B) Conformation 2 is characterized by a horizontal alignment of the side chain of F134W. This generates free space in the binding pocket, and intramolecular interactions of the substrate can be established.

While introducing various amino acids at positions D99, D304, and R330 led to more active enzyme variants, F134W was the only substitution that improved enzyme activity at position F134 (Figure 1D). The MD simulations revealed that the aromatic side chain of the residue at position F134 mainly occurs in two conformations: (I) vertical, pointing toward the active site (**Figure** **8**A and Table S12‐S15, Supporting Information), and (II) horizontal or vertical, pointing out of the enzyme (Figure 8B and Table S12‐S15, Supporting Information). We observed that the first conformation fixes the amide bond near the nucleophilic residue T267 and prevents the substrate from establishing intramolecular H‐bonds that causes the polymer chain to fold. Additionally, for variants harboring the F134W substitution, H‐bonds between the substrate and the indole side chain were established for up to 25% of the simulation time (Table S16, Supporting Information). Thereby, F134W can fix the amide bond of the substrate near the active residue T267, which may support substrate hydrolysis. Conformation 2 increases the free volume inside the binding pocket of the enzyme, allowing the substrate to move more freely and establish intramolecular H—bonds. We hypothesize that conformation 1 supports substrate hydrolysis and conformation 2 is responsible for substrate binding. As both conformations are essential for polymer degradation, it may explain why only a conservative substitution to an aromatic residue, which is similar in volume and shape to the wild‐type amino acid phenylalanine and thus can realize both conformations, leads to an active enzyme variant. Interestingly, a phenylalanine‐to‐tyrosine substitution was not observed during screening.

#### 2.3.5 Incremental Substrate Docking and MD Simulations Provide Insights into PA 6 Hydrolysis and the Structural Limitations of NylC‐type Enzymes

Based on the computational analysis, we attribute the positive effects on enzyme activity predominantly to the (I) reduction of steric and electrostatic interactions with the substrate, realized by the substitutions D99R and R330A, (II) elimination of unfavorable interactions, implemented by substitutions at position D304, and (III) formation of a H‐bond that fixes the amide bond near the active residue T267 by introducing F134W. In addition, the residue at position F134 appears to be crucial for opening/closing the binding pocket and preventing the substrate from establishing intramolecular interactions. The negative epistasis observed upon combining D99R with a substitution at position D304 is likely a result of the restoration of unfavorable enzyme–substrate interactions. The combination of incremental docking and subsequent MD simulations enabled targeted and efficient enzyme engineering. Moreover, MD simulations could be applied to generate understanding of changes in intra‐ (i.e., protein–protein and substrate–substrate) and intermolecular (i.e., enzyme–substrate) interactions.

The KnowVolution campaign yielded a polyamidase, NylC–HP, with the highest specific activity without improving the degree of depolymerization. Recently a promising strategy was presented where the degree of depolymerization of PETases could be enhanced. By increasing the flexibility of the PET‐binding groove of BhrPETase, it was possible to yield TurboPETase, which operates on versatile surface structures through dynamic binding.^[^ ^2^, ^51^ ^]^ Restructuring of the active site broadened the spectrum of attack sites accessible for enzymatic hydrolyzation and led to enzymes that nearly completely depolymerize PET at industrially relevant solids loading. Our simulations revealed that the active site of NylC–HP is located in a narrow pocket, which only offers space for a single PA 6 chain (Figure 3A). We hypothesize that NylC–HP can only hydrolyze exposed polymer chains, which explains the low degree of depolymerization (Figure 3C). To achieve a higher degree of depolymerization, the enzyme would need to strip PA chains from the polymer surface and break their intra‐ and interchain interactions before substrate binding to the relatively narrow binding pocket and subsequent hydrolysis can occur. Considering the low degree of depolymerization achieved, NylC–HP appears to be incapable of stripping off PA 6 chains that are firmly attached to the polymer. Finally, NylC–HP can cause surface alterations but cannot induce pitting and penetrate the inner polymer as, observed for PETases.^[^ ^10^, ^12^, ^39^, ^52^ ^]^ In comparison to TurboPETase, the active site of NylC–HP is more buried inside the enzyme. Therefore, a comprehensive redesign and substantial widening of the binding pocket in NylC–HP would be required to enhance the degree of PA 6 and PA 6,6 depolymerization.

## 3 Conclusion

Enzymatic recycling of synthetic polymers has emerged as a promising alternative to traditional recycling methods. However, environmentally friendly and cost‐effective polymer hydrolysis processes rely on stable enzymes with high activity and high degree of depolymerization. We engineered NylC_p2_–TS and developed the highest‐performing polyamidase to date, NylC–HP. Through the engineering of NylC_p2_–TS and in‐depth MD simulations of enzyme–substrate interactions, we identified key positions (D99, F134, D304, and R330) crucial for substrate interactions and hydrolysis, ultimately resulting in a 6.9‐fold improved *k*_cat_. Our study shows that the combination of incremental docking and MD simulation is an excellent guide for the efficient engineering of polyamidases with high specific activity while simultaneously generating molecular understanding. The incremental approach is particularly effective for engineering enzymes to process flexible polymers with numerous rotatable bonds, such as linear polyamides, ‐esters, and ‐urethanes. Interestingly, active site engineering did not result in any improvements of the degree of depolymerization. We conclude that large parts of PA 6 and PA 6,6 are not accessible for hydrolysis by NylC–HP, which could be attributed to an insufficient binding pocket size and/or flexibility. We anticipate that upcoming engineering campaigns will generate valuable insights, leading to strategies for remodeling binding pockets to enhance the degree of depolymerization for polyamidases. Moreover, novel polyamidases will be identified, which might represent a more promising starting point for engineering with a naturally broader active site. These can then be subjected to the general computational approach outlined in this study to enhance their activity.

## Conflict of Interest

The authors declare no conflict of interest.

## Supporting information

Supplementary Material

## Data Availability

The data that support the findings of this study are openly available in Zenodo at https://doi.org/10.5281/zenodo.11634510, reference number 11634510.
