# Supplementary material for: KnowVolution of an Efficient Polyamidase through Molecular Dynamics Simulations of Incrementally Docked Oligomeric Substrates
Source: ChemSusChem. 2025 Jun 26;18(15):e202500257. doi: 10.1002/cssc.202500257 (PMC12302326; doi:10.1002/cssc.202500257)
Supplement: Supplementary file 1 — Supplementary Material [file CSSC-18-e202500257-s001.pdf]

Supporting Information

KnowVolution Of An Efficient Polyamidase Through Molecular Dynamics Simulations Of Incrementally Docked Oligomeric Substrates

Hendrik Puetz[a], Alexander-Maurice Illig[a], Mariia Vorobii[b], Christoph Janknecht[a], Francisca Contreras[a], Fabian Flemig[a], Ulrich Schwaneberg*[a, b]

[a] Institute of Biotechnology
RWTH Aachen University
Worringer Weg 3, 52074, Aachen, Germany
E-mail: u.schwaneberg@biotec.rwth-aachen.de
[b] DWI-Leibniz Institute for Interactive Materials
Forckenbeckstraße 50, 52074, Aachen, Germany

Supporting information for this article is given via a link at the end of the document.

List of figures

Figure S1. Crystallinity and molecular weight analyses of PA 6 and PA 6,6. 13

Figure S2. Visualization of the enzyme-substrate complexes. 15

Figure S3. Conventional Michaelis-Menten kinetics of NylC$_{P2}$-TS, Single-substitutions and NylC$_{P2}$-TS$^{F134W/D304M}$ for PA 6. 18

Figure S4. Conventional Michaelis-Menten kinetics of NylC$_{P2}$-TS triple- and quadruple-substitutions for PA 6. 19

Figure S5. Conventional Michaelis-Menten kinetics of NylC$_{P2}$-TS and NylC-HP for PA 6,6. 20

Figure S6. HPLC Chromatogram of adipic acid, PA 6,6 monomer, Gf-PA 6,6 control, and Gf-PA 6,6 degradation NylC-HP. 20

Figure S7. Time-resolved Gf-PA 6 degradation by NylC$_{P2}$-TS and NylC-HP with high catalyst load. 21

Figure S8. Incremental procedure for docking Ace-[6-AHA]$_4$-COO$^-$ and H_3N^+-[6-AHA]$_4$-NMe to the receptor. 22

Figure S9 Incremental procedure for docking $^-$OOC-[6-AHA]$_4$-Ace and NMe-[6-AHA]$_4$-NH_3^+ to the receptor. 22

Figure S10. Root mean square deviation (RMSD) of the protein backbone of NylC$_{P2}$-TS. 23

Figure S11. Root mean square deviation (RMSD) of the protein backbone of NylC$_{P2}$-HP. 23

Figure S12. Root mean square deviation (RMSD) of the protein backbone of NylC-HP^{D99R}. 23

Figure S13. Root mean square deviation (RMSD) of the protein backbone of NylC$_{P2}$-TS^{D99R}. 24

List of Tables

Table S1. Percentage crystallinity (X_c) of untreated and heat-treated PA 6 and PA 6,6 determined by DSC. 14

Table S2. Molecular weight characteristics of commercial polymer samples as determined by GPC. 14

Table S3. Ligand poses ranked based on their affinity score towards NylC$_{P2}$-TS. 15

Table S4. Sequencing occurrence and specific activities of double substitutions from the iterative site-saturation mutagenesis of D304 with NylC$_{P2}$-TS^{F134W} as parental gene compared to NylC$_{P2}$-TS. 16

Table S5. Conventional Michaelis-Menten kinetics of NylC$_{P2}$-TS variants for PA 6. 17

Table S6. Conventional Michaelis-Menten kinetics of NylC$_{P2}$-TS variants for PA 6,6. 17

Table S7. Ligand poses ranked based on their affinity score towards NylC-HP. 24

Table S8. Ligand poses ranked based on their affinity score towards NylC-HP^{D99R}. 25

Table S9. Electrostatic interactions between substrate termini and residues D99R and R330 observed during MD simulation. 25

Table S10. Change in interaction energy upon H-bond formation between substrate termini and residues D99R and R330 observed during MD simulation. 26

Table S11. Electrostatic interactions between residues D191-R330 and D99R-D304 observed during MD simulation. 26

Table S12. Time of residue F134 being in distinct conformations for different systems containing NylC$_{P2}$-TS.......... 27

Table S13. Time of residue F134W being in distinct conformations for different systems containing NylC$_{P2}$-HP. 27

Table S14. Time of residue F134W being in distinct conformations for different systems containing NylC-HP^{D99R}. . 28

Table S15. Percentage of molecular dynamics (MD) simulation time of residue F134 (chain B) being in conformation 1 (Time$_{conf.\ 1}$) and conformation 2 (Time$_{conf.\ 2}$) for different systems containing NylC$_{P2}$-TS^{D99R}. 28

Table S16. H-bonds between the substrate and residue at position 134 observed during MD simulation. 29

Table S17. List of primers 30

Supplementary Materials and methods

Supplementary materials

All chemicals used in the experiments were of analytical grade or higher quality, sourced from Merck KGaA (Darmstadt, Germany), AppliChem GmbH (Darmstadt, Germany), and Carl Roth GmbH & Co. KG (Karlsruhe, Germany). The 6-AHA dimer was obtained from Toronto Research Chemicals Inc. (North York, Canada). Enzymes and reaction buffers were supplied by New England Biolabs (Frankfurt, Germany). Oligonucleotides were acquired from Eurofins MWG Operon Inc. (Ebersberg, Germany). Plasmid extraction and PCR purification were carried out using the NucleoSpin™ Plasmid Extraction and NucleoSpin™ Gel and PCR Clean-up Kits from Macherey-Nagel GmbH & Co. KG (Düren, Germany). Gf-PA 6 film (0.2 mm thick, AM30-FM-000200) and Gf-PA 6,6 granules (3 mm in diameter, AM32-GL-000115) were acquired from Goodfellow GmbH (Hamburg, Germany). For the Gf-PA 6 film, 6.4 mm diameter discs (7.6 mg each) were punched using a 12 kN 96-well puncher (Gechter, Germany).

Nucleotide and amino acid sequences of applied enzymes

Introduced mutations are underlined and in bold.

NylC$_{p2}$-TS nucleotide sequence:

ATGATGCATCATCATCATCATCACGGGGCAGGTGCCAATACCACACCGGTTCATGCACT
GACCGATATTGATGGTGGTATTGCAGTTGATCCGGCACCGCGTCTGGCAGGTCCGCCT
GTTTTTGGTGGTCCGGGTAATGCTGCATTCGATCTGGCACCGGTTCGTAGCACCGGTC
GTGAAATGCTGCGTTTTGATTTTCCGGGTGTTAGCATTGGTGCAGCACATTATGAAGAA
GGTCCGACAGGCGCAACCGTTATTCATATTCCGGCAGGCGCACGTACCGCAGTTGATG
CACGTGGTGGTGCAGTTGGTCTGAGCGGTGGTTATGATTTTAATCATGCAATTTGCCTG
GCAGGCGGTGCAGGTTATGGTCTGGAAGCCGGTGCCGGTGTTAGTGGTGCACTGCTG
GAACGTCTGGAATATCGTACCGGTTTTGCAGAACTGCAGCTGGTTAGCAGCGCAGTTAT
CTATGATTTTTCAGCACGTTCAACCGCAGTTTATCCTGATAAAGCACTGGGTCGTGCAG
CACTGGAATTTGCAGTTCCGGGTGAATTTCCGCAGGGTCGTGCCGGTGCGGGTATGAG
CGCAAGCGCAGGTAAAGTTGATTGGGATCGTACCGAAATTACCGGTCAGGGTGCAGCC
TTTCGTCGTCTGGGTGATGTTCGTATTCTGGCAGTTGTTGTTCCGAATCCGGTTGGTGT
TATTGTTGATCGTGCAGGCACCGTTGTTCGTGGTAATTATGATGCACAGACCGGTGTTC
GTCGTCATCCGGTTTTTGATTATCAAGAAGCATTTGCCGAACAGGTTCCTCCGGTTACC
CAAGCAGGTAATACCACAATTAGCGCCATTGTTACCAATGTGCGTATGAGTCCGGTTGA
ACTGAATCAGTTTGCGAAACAGGTTCATAGCAGCATGCATCGTGGCATTCAGCCGTTTC
ATACAGATATGGATGGTGATACCCTGTTTGCAGTTACCACCGATGAAATTGATCTGCCG

ACAACACCGGGTAGCAGCCGTGGTCGTCTGAGCGTTAATGCAACCGCACTGGGTGCAA
TTGCCAGCGAAGTTATGTGGGATGCCGTTCTGGAAGCGGGTAAATAA

NylC$_{p2}$-TS amino acid sequence:

MMHHHHHHGA[1]GANTTPVHALTDIDGGIAVDPAPRLAGPPVFGGPGNAAFDLAPVRSTGR
EMLRFDFPGVSIGAAHYEEGPTGATVIHIPAGARTAVDARGGAVGLSGGYDFNHAICLAGG
AGYGLEAGAGVSGALLERLEYRTGFAELQLVSSAVIYDFSARSTAVYPDKALGRAALEFAVP
GEFPQGRAGAGMSASAGKVDWDRTEITGQGAAFRRLGDVRILAVVVPNPVGVIVDRAGTV
VRGNYDAQTGVRRHPVFDYQEAFAEQVPPVTQAGNTTISAIVTNVRMSPVELNQFAKQVH
SSMHRGIQPFHTDMDGDTLFAVTTDEIDLPTTPGSSRGRLSVNATALGAIASEVMWDAVLE
AGK*

NylC-HP nucleotide sequence:

ATGCATCATCATCATCATCACGGGGCAGGTGCCAATACCACACCGGTTCATGCACTGA
CCGATATTGATGGTGGTATTGCAGTTGATCCGGCACCGCGTCTGGCAGGTCCGCCTGT
TTTTGGTGGTCCGGGTAATGCTGCATTCGATCTGGCACCGGTTCGTAGCACCGGTCGT
GAAATGCTGCGTTTTGATTTTCCGGGTGTTAGCATTGGTGCAGCACATTATGAAGAAGG
TCCGACAGGCGCAACCGTTATTCATATTCCGGCAGGCGCACGTACCGCAGTTGATGCA
CGTGGTGGTGCAGTTGGTCTGAGCGGTGGTTATGATTTTAATCATGCAATTTGCCTGGC
AGGCGGTGCAGGTTATGGTCTGGAAGCCGGTGCCGGTGTTAGTGGTGCACTGCTGGA
ACGTCTGGAATATCGTACCGGT**<u>TGG</u>**GCAGAACTGCAGCTGGTTAGCAGCGCAGTTATC
TATGATTTTTCAGCACGTTCAACCGCAGTTTATCCTGATAAAGCACTGGGTCGTGCAGC
ACTGGAATTTGCAGTTCCGGGTGAATTTCCGCAGGGTCGTGCCGGTGCGGGTATGAGC
GCAAGCGCAGGTAAAGTTGATTGGGATCGTACCGAAATTACCGGTCAGGGTGCAGCCT
TTCGTCGTCTGGGTGATGTTCGTATTCTGGCAGTTGTTGTTCCGAATCCGGTTGGTGTT
ATTGTTGATCGTGCAGGCACCGTTGTTCGTGGTAATTATGATGCACAGACCGGTGTTCG
TCGTCATCCGGTTTTTGATTATCAAGAAGCATTTGCCGAACAGGTTCCTCCGGTTACCC
AAGCAGGTAATACCACAATTAGCGCCATTGTTACCAATGTGCGTATGAGTCCGGTTGAA
CTGAATCAGTTTGCGAAACAGGTTCATAGCAGCATGCATCGTGGCATTCAGCCGTTTCA
TACA**<u>ATG</u>**ATGGATGGTGATACCCTGTTTGCAGTTACCACCGATGAAATTGATCTGCCGA
CAACACCGGGTAGCAGCCGTGGT**<u>GCG</u>**CTGAGCGTTAATGCAACCGCACTGGGTGCAA
TTGCCAGCGAAGTTATGTGGGATGCCGTTCTGGAAGCGGGTAAA

NylC-HP amino acid sequence:

MMHHHHHHGA[1]GANTTPVHALTDIDGGIAVDPAPRLAGQPVFGGPGNAAFDLAPVRSTGR
EMLRFDFPGVSIGAAHYEEGPTGATVIHIPAGARTAVDARGGAVGLSGGYDFNHAICLAGG
AGYGLEAGAGVSGALLERLEYRTG**<u>W</u>**AELQLVSSAVIYDFSARSTAVYPDKALGRAALEFAV
PGEFPQGRAGAGMSASAGKVDWDRTEITGQGAAFRRLGDVRILAVVVPNPVGVIVDRAGT
VVRGNYDAQTGVRRHPVFDYQEAFAEQVPPVTQAGNTTISAIVTNVRMSPVELNQFAKQV

HSSMHRGIQPFHT**M**MDGDTLFAVTTDEIDLPTTPGSSRG**A**LSVNATALGAIASEVMWDAVL EAGK*

Supplementary experimental methods

4.2.1 Gene construction

The gene encoding the parental enzyme, $NylC_{p2}$-TS, was generated by the commercial synthesis of the $NylC_{p2}$ wildtype by Eurofins, cloning into pET 21a (+) (Novagen), and introduction of four amino acid substitutions to obtain the thermostable $NylC^{D36A/D122G/H130Y/E263Q}$ ($NylC_{p2}$-TS).[1]

Library construction

Site-saturation mutagenesis (SSM) libraries of $NylC_{p2}$-TS were generated using NNK primers (**Table S17**). Polymerase chain reaction (PCR) was conducted using Q5® High-Fidelity DNA Polymerase (New England BioLabs, USA). PCR products were incubated with DpnI (New England BioLabs, USA; 37 °C, 60 min) to digest template DNA, purified, and transformed into chemically competent *E. coli* BL21(DE3) cells.

Library cultivation

SSM libraries were cultivated in 96-well F-bottom MTPs (Greiner, Frickenhausen, Germany) by inoculating 200 µL lysogeny broth media (LB media, 1 % tryptone, 0.5 % yeast extract, and 1 % NaCl, 50 µg mL^{-1} carbenicillin) with a single freshly transformed colony per well. 12 wells were inoculated with $NylC_{p2}$-TS as parental enzyme reference. After overnight incubation (900 RPM, 37 °C, 70 % humidity, 16 h), 10 µL pre-culture were added to 130 µL Terrific broth media (TB media, 24 g L^{-1} yeast extract, 20 g L^{-1} tryptone, 4 mL L^{-1} glycerol, 0.017 M KH_2PO_4, 0.072 M K_2HPO, pH 7.4, 50 µg mL^{-1} carbenicillin) in 96-well V-bottom MTPs. Following growth to an OD_{600} of 0.6 (900 RPM, 37 °C, 70 % humidity, 2 h), expression was induced by the addition of 10 µL isopropyl-β-D-thiogalactopyranoside (IPTG, 1.5 mM). Cultures were further incubated overnight (900 RPM, 20 °C, 70 % humidity, 20 h) and harvested by centrifugation (Eppendorf 5810R; 3,220 x *g*, 4 °C, 15 min). The obtained cell pellet was resuspended with 150 µL buffer (50 mM Bicine, pH 8.0, 100 mM NaCl, 20 °C, 10 min) to remove the residual medium and pelleted again. Cells were disrupted by resuspension and incubation in 150 µL lysis buffer (50 mM Bicine, pH 8.0, 1.5 mg mL^{-1} lysozyme, 25 µg mL^{-1} DNAseI; 900 RPM, 37 °C, 2 h). Insoluble cell residue was pelleted (Eppendorf 5810R; 3,220 x *g*, 4 °C, 15 min) and the cell-free extract was used for the screening.

Library screening

Screening for improved enzyme variants was conducted using the AMIDE method.[2] Screening reactions were initiated by adding 10 µL of clarified cell lysate to 190 µL screening buffer (50 mM Bicine, pH 9.0, 100 mM NaCl) containing 38 g L^{-1} Gf-PA 6 film in a 96-well F-bottom MTP. Before incubation (800 RPM, 60 °C, 4 h), plates were sealed (EasySeal™, Greiner GmbH, Germany) to prevent evaporation. Plates were then centrifuged to precipitate denatured protein (Eppendorf 5810R, 4 °C, 3,220 x *g*, 20 min), and 100 µL of the reaction mixture was analyzed via MAFC assay.

MAFC assay

Released amines were detected by MAFC MTP assay. To prepare the MAFC assay solution, 4 mM Meldrum's acid furfural conjugate (MAFC) were dissolved in 100 % ethanol and filtered through a 0.45 µm syringe filter (Aerodiscs®, Pall Deutschland Holding GmbH & Co. KG, Germany). Next, plates were developed (800 RPM, 37 °C, 30 min), centrifuged (Eppendorf 5810R, 3,220 x *g*, 4 °C, 1 min), and the absorbance was measured at 494 nm (CLARIOstar, BMG LABTECH, Germany; 100 flashes/well).

High-performance liquid chromatography (HPLC) analysis of PA degradation products

PA 6 and PA 6,6 degradation products (6-aminohexanoic acid, adipic acid, PA 6 dimer, and PA 6,6 dimer) were analyzed using a Shimadzu prominence LC system together with a C18-RP column (NUCLEOSIL 100-5 C18, Macherey-Nagel GmbH & Co. KG, Germany). Analytes were quantified via a photodiode array (Nexera X2 SPD-M30A, Shimadzu, Japan) at 220 nm. Mobile phase A comprised 0.1 % sulfuric acid and mobile phase B comprised 100 % acetonitrile. Analyte injections of 10 µL were isocratically resolved. For PA 6, 5 % mobile phase B with a constant flow rate of 1 mL min^{-1} over a total runtime of 20 min was applied. For PA 6,6 15 % mobile phase B with a constant flow rate of 1 mL min^{-1} over a total runtime of 20 min was applied.

Preparative scale enzyme expression and purification

Selected $NylC_{p2}$-TS variants were expressed in *E. coli* BL21 (DE3). Induction was carried out with 0.1 mM IPTG at an OD600 of 0.6, followed by a 20-hour expression at 18 °C. Cells were collected by centrifugation (3,220 x g, 20 min, 4 °C). The resulting pellet was resuspended in lysis buffer (50 mM NaH_2PO_4, pH 8.0, 300 mM NaCl) containing 1.5 mg/mL lysozyme and incubated for 30 min at 37 °C. Cell disruption was achieved through sonication (Vibra-Cell™ VCX 130, Sonics & Materials Inc., USA, 5 x 30 s bursts with 30 s cooling intervals, 60 %

amplitude). The lysate was centrifuged (Eppendorf 5810R, 10,000 RPM, 30 min, 4 °C), filtered through a 0.45 μm syringe filter, and loaded onto a Ni-IDA 2000 column (Macherey-Nagel GmbH & Co. KG, Düren, Germany). Unspecifically bound proteins were washed away with 30 mL lysis buffer, and the target protein was eluted with 2.5 mL elution buffer (50 mM NaH_2PO_4, pH 8.0, 300 mM NaCl, 250 mM imidazole). The buffer was exchanged for storage buffer (50 mM Bicine, pH 8.0, 100 mM NaCl) using PD-10 columns (Cytiva, MA, USA). The protein was then concentrated using ultra-centrifugal filter units (10 kDa Amicon®, Merck KGaA, Darmstadt, Germany) and stored at −20 °C after snap freezing in liquid nitrogen. Protein purity was verified by sodium dodecyl sulfate-polyacrylamide gel electrophoresis (SDS-PAGE), and protein concentrations were measured using the Bradford method (Pierce™ Coomassie Plus, ThermoFisher Scientific, Wesel, Germany).[3]

Michaelis-Menten kinetics

To determine the maximum reaction velocity, enzyme activity was assayed at enzyme saturation (Gf-PA 6 film, 38–684 g L^{-1}; Gf-PA 6,6 granules, 110–880 g L^{-1}), and the curves were fitted using the Hill equation (**Equation 1**) with v being the enzymatic reaction rate observed at a particular substrate concentration, V_{max} the maximum reaction velocity, [S] the substrate concentration, K the dissociation constant, representing the substrate concentration at which half of the binding sites are occupied, and $n = 2$ as fixed Hill coefficient using OriginPro® 2024 (OriginLab). Kinetic parameters were derived from the Michaelis-Menten Equation (**Equation 2**), with v being the enzymatic reaction rate observed at a particular substrate concentration, V_{max} the maximum reaction velocity, [S] the substrate concentration, and K_M the Michaelis-Menten constant.

$$v = \frac{V_{\max}[S]^n}{K^n + [S]^n} \tag{S1}$$

$$v = V_{\max}\frac{[S]}{K_{\mathrm{M}}[S]} \tag{S2}$$

Melting temperature analysis

Enzyme melting temperatures (T_m) of $NylC_{p2}$-TS variants were measured using nano-Differential Scanning Fluorimetry (nanoDSF) using a Prometheus NT.48 device from NanoTemper Technologies GmbH (München, Germany). Measurements were conducted at a fluorescence intensity of 45 %, with the temperature range set from 20 °C to 95 °C and a heating rate of 1 °C per minute.

PA crystallinity analysis by differential scanning calorimetry (DSC)

The percentage crystallinity of the PA 6 film and PA 6,6 granules was determined by DSC without and after heat treatment at different temperatures ranging from 40 to 90 °C in 10 °C increments. Prior to DSC measurement, all tested samples were dried under a vacuum overnight to remove absorbed water. Then, samples were weighed (approx. 7.4 mg for PA 6 and 10–12 mg for PA 6,6) and sealed in 50 µL aluminum pans (Perkin Elmer, part No. BO143017) with covers (Perkin Elmer, part No. BO143003). Covers were punctured (3 punctures ~0.2 mm in diameter), and encapsulated pans were weighed before and after DSC runs. DSC measurements were performed on Perkin Elmer Differential Scanning Calorimeter DSC 8500. The instrument was calibrated for temperature and heat flow using indium standards prior to the analysis to ensure accuracy. Samples were equilibrated at 0 °C, then heated to 270 °C for PA 6 and to 310 °C for PA 6,6 at a rate of 10 °C min^{-1}, and cooled down by using a −50 °C cooling system, Perkin Elmer controlled Liquid Nitrogen Accessory CLN2. The area under the endothermic peak corresponding to the melting of PA 6 and PA 6,6 was integrated to determine the heat of fusion (ΔH_{m}) of the sample using Pyris Software, Version 13.3.1.0014. The crystallinity percentage was calculated using equation (S3).

$$\%\ \mathrm{Crystallinity} = \left(\frac{\Delta H_{\mathrm{m}}}{\Delta H_{\mathrm{m}}^{0}}\right) \times 100\% \qquad \text{Eq. (S3)}$$

Where $\Delta H_{\mathrm{m}}^{0}$ is the heat of fusion for 100% crystalline PA 6 (230 J g^{-1}), and PA 6,6 (190 J g^{-1}).[4]

PA molecular weight analysis by gel permeation chromatography (GPC)

Molecular weights (the number-average molecular weight (M_{n}) and the weight-average molecular weight (M_{w})) and dispersity ($Đ_{\mathrm{M}}$) were determined by GPC. The GPC analyses were carried out using 1,1,1,3,3,3-hexafluoro-2-propanol (HFIP) (99.9 %, Chempur) as eluent, supplemented with 0.05 mol L^{-1} sodium trifluoracetate (NaTFAc) (≥ 98 %, Thermo Scientific). Each investigated polymer sample was dissolved in HFIP at a concentration of 1 mg·mL^{-1}.

The analytical setup is equipped with an HPLC pump (1200, Agilent), a refractive index detector (RI) (1200, Agilent), and a UV-detector (VWD, 1200, Agilent). The samples contained 250 mg L^{-1} 3,5-di-*tert*-4-butylhydroxytoluene (BHT, ≥99 %, Fluka) as internal standard. One pre-column (4.6 × 50 mm) and two PFG gel columns (4.6 × 250 mm, Polymer Standards Service) were applied at a flow rate of 0.3 mL min^{-1} at 40 °C. The gel particles had a diameter of 5 µm, with nominal pore widths of 10^2 and 10^3 Å. Calibration was performed using narrowly distributed poly(methyl methacrylate) standards (Polymer Standards Service). The data analysis was conducted using the PSS WinGPC UniChrom software (Version 8.3.2).

Chemical synthesis of MAFC

MAFC was synthesized as previously reported.[5] Briefly, 1.51 g (10.5 mmol) of 2,2-dimethyl-1,3-dioxane-4,6-dione was mixed with 961 mg (10 mmol) of 2-furaldehyde in 30 mL water and stirred at 70 °C for 2 h. The product precipitates as yellow solid and was dissolved in dichloromethane, washed with saturated aqueous $NaHSO_3$, H_2O, $NaHCO_3$, and brine (30 mL each), and filtered. Finally, organic solvents were evaporated to obtain a bright yellow powder.

Supplementary Computational Methods

Incremental docking

Enzyme (receptor) preparation

First, the structure of the 6-aminohexanoate-oligomer hydrolase $NylC_{p2}$-TS (PDB identifier 5Y0M) was fetched from the RCSB Protein Data Bank.[6-7] Heteroatoms were removed and symmetry mates were generated to obtain the tetrameric structure using PyMOL™ Molecular Graphics System (version 2.3.0, Schrödinger, LLC). The missing residues V29 and F30 were modeled with MODELLER (version 10.4).[8] During this procedure the non-missing residues were fixed. Further, MODELLER was used to generate enzyme variants by introducing the variant-specific substitutions. The N-terminal residues D19 and I16 were capped with an acetyl group (Ace), while an N-methyl (NMe) cap was used for the C-terminal residues P259 and P260. The protonation state of the enzyme was adjusted according to a pH value of 8.0.

Energy minimization was performed with GROMACS (version 2022.3) using the CHARMM36 force field (version July 2022) and the TIP3P water model.[9-11] For energy minimization, the system was solvated, neutralized, and a concentration of 100 mM NaCl was adjusted. We applied the „split the charge difference in two“ rule to add proper amounts of ions.[12] For energy minimization, the steepest descent algorithm was applied until a maximum force of 1000 kJ mol^{-1} nm^{-1} on any atom was reached. The energy-minimized enzyme structure was extracted and receptor preparation was performed with AutoDockFR (version 1.2rc1).[13] The substituted residues at positions D99, F134, D304, and R330 were treated as flexible during the docking procedure.

Substrate (ligand) preparation

We used Ace-$[6\text{-AHA}]_4$-COO^- and NMe-$[6\text{-AHA}]_4$-NH_3^+ oligomers as model substrates to mimic the PA 6 chain and study the enzyme-substrate interactions (Error! Reference source not found.).

The substrates (fragments) were generated and energy minimized with AVOGADRO (version 1.2.0).[14] Substrate preparation was performed with AutoDockFR (version 1.2rc1).[13]

Incremental docking procedure

To overcome the sampling problem when performing docking experiments with substrates that have many rotatable bonds, we implemented and applied a parallelized, incremental meta-docking procedure inspired by DINC. [15-17] Our workflow can be summarized as follows:

1. Select Ace-[6-AHA]-NMe as the first fragment to be docked to the prepared enzyme structure.
2. To ensure broad conformation sampling and efficient use of computing resources, we performed 240 parallel runs of AutoDock Vina (version 1.2.5) to generate a pool of fragment poses.[18-19] The generated conformations were clustered based on their root mean square deviation using a cutoff of 1.5 nm and structures that presented the bond to be cleaved near the active residue T267 were selected for the next round.
3. The selected fragments of the previous round were extended by another unit of 6-AHA and the bonds of the parental fragment were set to be non-rotatable. The structure was energy minimized with AVOGADRO (version 1.2.0) and prepared for docking with AutoDockFR (version 1.2rc1).[13] After extension and preparation, conformation sampling was executed as described in 2.
4. Iterative cycles of 2 and 3 were performed until the structure consisted of four units of 6-AHA.To avoid bias, we performed fragment extension such that both orientations of each substrate (i.e., Ace-[6-AHA]$_4$-COO$^-$ and NMe-[6-AHA]$_4$-NH$_3^+$) were generated (**Figure S8-S9**). The final poses were clustered and evaluated based on their affinity and population. Poses that had a high population and a low free energy of binding were selected for molecular dynamics (MD) simulation.

Molecular dynamics simulation

MD simulations were performed to study the dynamic behavior of different enzyme variants and enzyme-substrate complexes. At first, parameters were assigned to the substrates using the CHARMM general force field program (version 2.5).[20-21] MD simulations were performed with GROMACS (version 2022.3) using the CHARMM36 force field (version July 2022) and the TIP3P water model.[9-11] Hydrogens were added to the side chains to mimic a pH value of 8.0. The enzyme(-substrate complex) was centered in a cubic box with 1 nm between the solute and the box. The system was solvated, neutralized, and a concentration of 100 mM NaCl was adjusted. To ensure neutralization while not exceeding the concentration of 100 mM NaCl, we applied the „split the charge difference in two“ rule.[12] Energy minimization was performed applying the steepest descent algorithm until a maximum force of 1000 kJ mol^{-1} nm^{-1} on any atom was reached. The system was equilibrated by a 1 ns *NVT* run at 293.15 K using the modified Berendsen thermostat (velocity rescaling), while coupling protein(/substrate) and water/ions separately to the temperature bath using a coupling constant of 0.1 ps, followed by a 1 ns *NpT* run at 293.15 K and 1.013 bar using the previously described modified Berendsen thermostat and the Parinello-Rahman barostat with the following parameters:

pcoupltype = isotropic, tau_p = 1.0 ps, and compressibility = 4.5 · 10^{-5}. For equilibration, position restraints were applied to the protein (and the substrate) using the linear constraint solver (LINCS) algorithm. To generate simulation data, the position restraints were removed, and a 150 ns production run was performed under *NpT* conditions for the enzyme-substrate complexes and a 200 ns run was performed for the enzyme-only systems. The leap-frog algorithm was used with an integration time of 2 fs for solving Newton‘s equations of motion. Particle-Mesh Ewald electrostatics were used to calculate the Coulomb interactions with a short-range electrostatic and a van der Waals cutoff of 1.0 nm. Three independent MD runs were performed for each system. The first 20 ns of the simulation were not considered for analysis (**Figure S10-S13**).

Conservation analysis

First, a protein-protein BLAST was run to extract a set of similar sequences from the non-redundant protein sequences database (update date 2024/06/10) using the sequence of $NylC_{p2}$-TS (PDB identifier 5Y0M) as query.[6, 22] The maximum number of aligned sequences to display was increased to 5000, while the other algorithm parameters remained unchanged. Next, a multiple sequence alignment was constructed with Clustal Omega (clustalo version 1.2.4) using sequences with an *E*-value ≤ 1 · 10^{-100} and a sequence identity ≤ 95 %.[23-25] The sequence logo was generated according to the procedure described by Schneider et al.[26]

Supplementary figures and tables

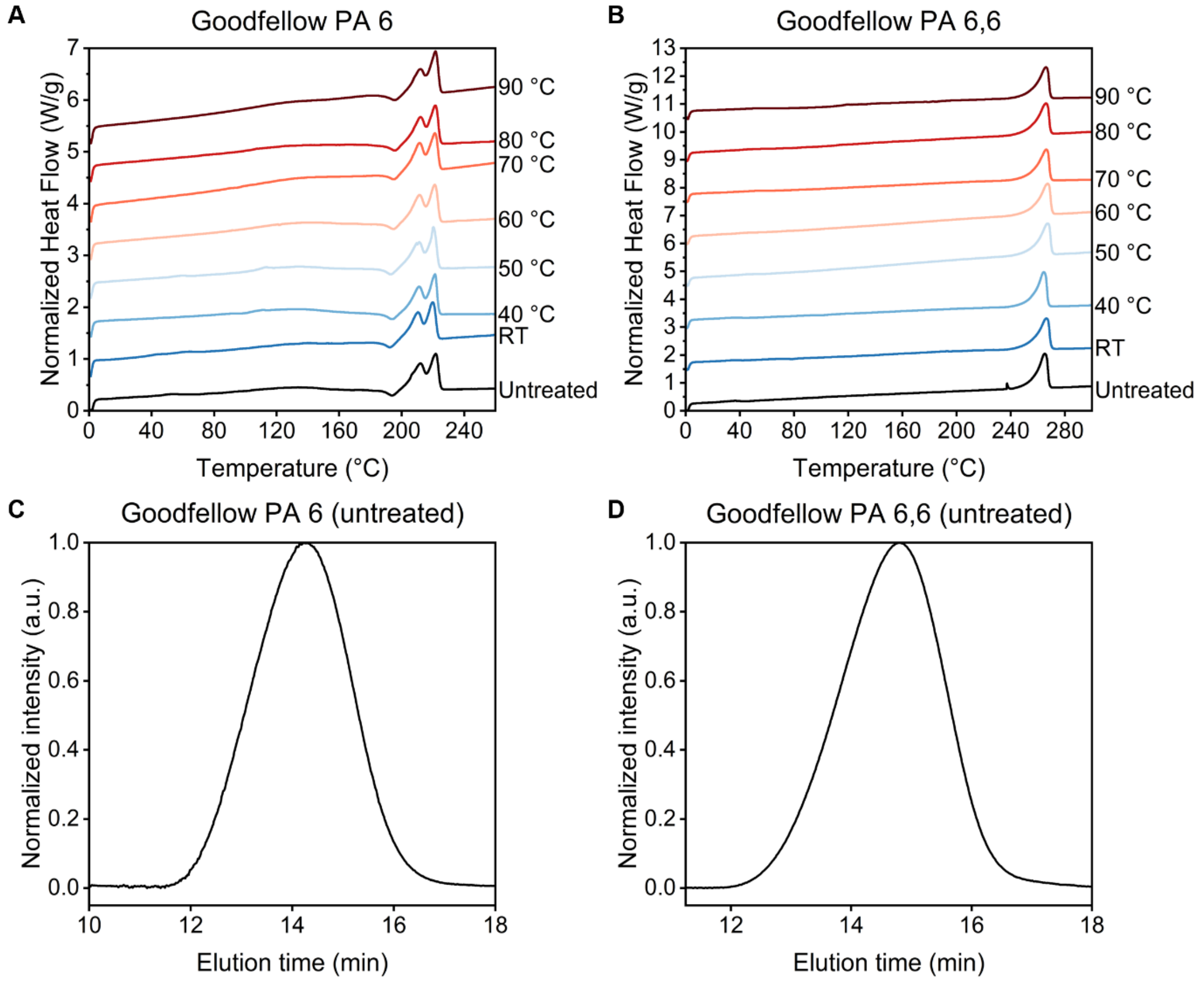

Figure S1. Crystallinity and molecular weight analyses of PA 6 and PA 6,6.

A) DSC thermograms of untreated and heat-treated PA 6 samples. PA 6 samples were either analyzed as provided (untreated), or pre-incubated in buffer (50 mM bicine, pH 8.0, 100 mM NaCl) at 30–90 °C for 24 h. B) Normalized GPC elugram (HFIP) of untreated Goodfellow PA 6 film (0.2 mm thick). C) Normalized GPC elugram (HFIP) of untreated Goodfellow PA 6,6 granules (3 mm diameter).

Table S1. Percentage crystallinity (X_c) of untreated and heat-treated PA 6 and PA 6,6 determined by DSC.

**PA 6**			**PA 6,6**		
Sample	ΔH_m (J g^{-1})	% Crystallinity	Sample	ΔH_m (J g^{-1})	% Crystallinity
Untreated	49	21	Untreated	75	39
RT	50	22	RT	76	40
40 °C	50	22	40 °C	74	39
50 °C	51	22	50 °C	74	39
60 °C	50	22	60 °C	74	39
70 °C	50	22	70 °C	74	39
80 °C	50	22	80 °C	74	39
90 °C	50	22	90 °C	74	39

Table S2. Molecular weight characteristics of commercial polymer samples as determined by GPC.

This table presents the molecular weight characteristics, including number-average molecular weight (M_n), weight-average molecular weight (M_w), and dispersity index ($Đ_M$), of various commercial polymer samples.

**Sample**	**M_n (Da)**	**M_w (Da)**	**$Đ_M$**
Good fellow PA 6 film, 0.2 mm thickness	44520	108500	2.438
Good fellow PA 6,6 granules, 3°mm diameter	29300	61840	2.111

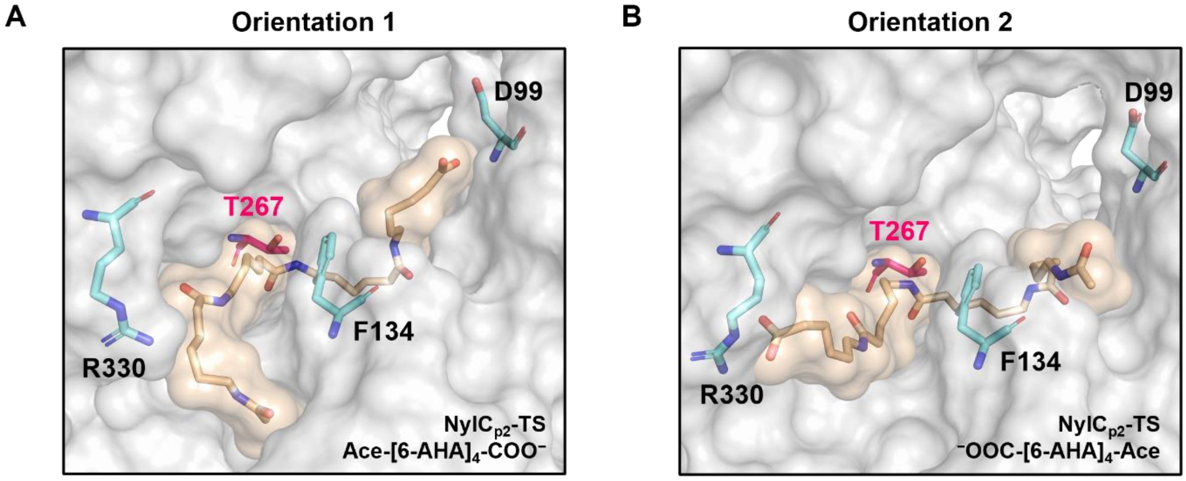

Figure S2. Visualization of the enzyme-substrate complexes.

A) NylC$_{p2}$-TS/Ace-[6-AHA]$_4$-COO$^-$ and B) NylC$_{p2}$-TS/$^-$OOC-[6-AHA]$_4$-Ace. As shown, the sequence of the termini (i.e., Ace and COO$^-$) used for substrate naming refers to the orientation of the substrate inside the pocket. The residues D99, F134, T267, and R330 are highlighted as reference points. The naming of the substrates NMe-[6-AHA]$_4$-NH_3^+ and H_3N^+-[6-AHA]$_4$-NMe follows the same logic.

Table S3. Ligand poses ranked based on their affinity score towards NylC$_{p2}$-TS.

Calculations were performed with AutoDock Vina (version 1.2.5).[18-19] Incremental docking of PA 6 model substrates starting from Ace-[6-AHA]-NMe and NMe-[6-AHA]-Ace revealed that the substrates Ace-[6-AHA]$_4$-COO$^-$ and $^-$OOC-[6-AHA]$_4$-Ace generate the most favorable poses (i.e., poses with low affinity scores and high populations). In this context, 6-AHA refers to 6-aminohexanoic acid, Ace to an acetyl cap, and NMe to an N-Methyl cap.

	**Ace-[6-AHA]$_4$-COO$^-$**		**$^-$OOC-[6-AHA]$_4$-Ace**	
**Rank**	Affinity (kcal mol^{-1})	Occurrences out of 1000	Affinity (kcal mol^{-1})	Occurrences out of 1000
1	−13.27 ± 0.01	63	−13.81 ± 0.03	23
2	−13.25 ± 0.01	82	−13.71 ± 0.04	20
3	−13.23 ± 0.01	55	−13.59 ± 0.02	29
4	−13.22 ± 0.01	82	−13.58 ± 0.02	29
5	−13.21 ± 0.01	71	−13.55 ± 0.03	21
6	−13.16 ± 0.02	24	−13.48 ± 0.02	27
7	−13.15 ± 0.01	78	−13.47 ± 0.02	23
8	−13.13 ± 0.02	27	−13.42 ± 0.02	20
9	−13.09 ± 0.01	59	−13.36 ± 0.01	23
10	−13.06 ± 0.01	38	−13.33 ± 0.01	21

Table S4. Sequencing occurrence and specific activities of double substitutions from the iterative site-saturation mutagenesis of D304 with NylC$_{p2}$-TS^{F134W} as parental gene compared to NylC$_{p2}$-TS.

Errors represent the standard error of mean.

Substitution F134W/D304X	Occurrence	Specific activity ($\mu mol_{6\text{-}AHAeq.}\ h^{-1}\ mg_{enzyme}^{-1}$)	Improvement (fold)
M	6 (13 %)	410 ± 10	5.3 ± 0.1
S	6 (13 %)	223 ± 17	2.9 ± 0.2
E	5 (11 %)	239 ± 7	3.1 ± 0.1
I	5 (11 %)	341 ± 5	4.4 ± 0.1
L	5 (11 %)	371 ± 17	4.8 ± 0.2
R	5 (11 %)	248 ± 12	3.2 ± 0.2
V	4 (9 %)	202 ± 10	2.6 ± 0.1
C	3 (7 %)	n.d.	n.d.
A	2 (4 %)	n.d.	n.d.
Q	1 (2 %)	n.d.	n.d.
F	1 (2 %)	n.d.	n.d.
T	1 (2 %)	n.d.	n.d.
D (NylC$_{p2}$-TS^{F134W})	1 (2 %)	217 ± 5	2.8 ± 0.1

Table S5. Conventional Michaelis-Menten kinetics of NylC$_{p2}$-TS variants for PA 6.

Variant					Conc. (nM)	V_{max} (µM s^{-1})	k_{cat} (s^{-1})	Specific activity (µmol$_{6\text{-AHAeq.}}$ h^{-1} mg$_{enzyme}$$^{-1}$)	Improv. (fold)
**NylC$_{p2}$-TS**					**50**	**0.040 ± 0.002**	**0.79 ± 0.03**	**75 ± 3**	**1.0**
	D99R				50	0.114 ± 0.005	2.27 ± 0.11	216 ± 10	2.9
		F134W			50	0.118 ± 0.002	2.36 ± 0.05	224 ± 4	3.0
			D304M		50	0.130 ± 0.001	2.60 ± 0.02	247 ± 2	3.3
				R330A	50	0.077 ± 0.000	1.55 ± 0.01	147 ± 1	2.0
		F134W	D304M		25	0.115 ± 0.002	4.60 ± 0.08	437 ± 7	5.8
V4	D99R	F134W	D304M		25	0.065 ± 0.001	2.61 ± 0.03	248 ± 3	3.3
V3	D99G	F134W	D304M		25	0.070 ± 0.002	2.80 ± 0.06	274 ± 6	3.6
V5	D99V	F134W	D304M		25	0.053 ± 0.002	2.12 ± 0.07	199 ± 6	2.7
**NylC-HP (V1)**		**F134W**	**D304M**	**R330A**	**25**	**0.137 ± 0.005**	**5.48 ± 0.20**	**520 ± 19**	**6.9**
V2		F134W	D304M	R330Q	25	0.098 ± 0.005	3.92 ± 0.20	371 ± 19	4.9
V8	D99R	F134W	D304M	R330A	25	0.078 ± 0.001	3.12 ± 0.05	296 ± 5	3.9
V6	D99G	F134W	D304M	R330A	25	0.108 ± 0.006	4.32 ± 0.24	410 ± 23	5.5
V7	D99V	F134W	D304M	R330A	25	0.103 ± 0.005	4.12 ± 0.20	391 ± 19	5.2
V10	D99R	F134W	D304M	R330Q	25	0.067 ± 0.011	2.68 ± 0.44	255 ± 42	3.4
V9	D99G	F134W	D304M	R330Q	25	0.068 ± 0.005	2.72 ± 0.20	258 ± 19	3.4
V11	D99V	F134W	D304M	R330Q	25	0.054 ± 0.002	2.16 ± 0.08	205 ± 8	2.7

Table S6. Conventional Michaelis-Menten kinetics of NylC$_{p2}$-TS variants for PA 6,6.

Variant				Conc. (nM)	V_{max} (µM s^{-1})	k_{cat} (s^{-1})	Specific activity (µmol$_{PA6,6\ monomer\ eq.}$ h^{-1} mg$_{enzyme}$$^{-1}$)	Improv. (fold)
**NylC$_{p2}$-TS**				**20**	**0.064 ± 0.002**	**3.20 ± 0.1**	**304 ± 9**	**1.0**
**NylC-HP**	**D99R**	**F134W**	**R330A**	**7.5**	**0.081 ± 0.002**	**10.8 ± 0.3**	**1026 ± 25**	**3.4**

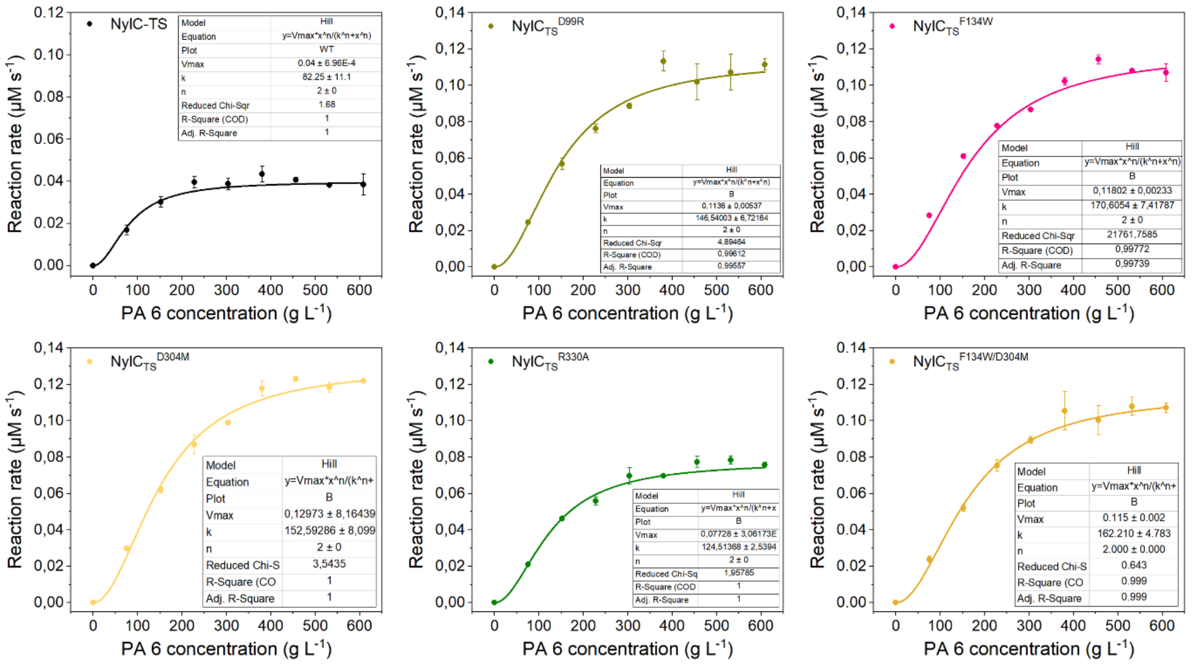

Figure S3. Conventional Michaelis-Menten kinetics of NylC$_{p2}$-TS, Single-substitutions and NylC$_{p2}$-TS$^{F134W/D304M}$ for PA 6.

^{Conv}MM kinetics were conducted at enzyme saturation (50 nM/1.90 mg L^{-1} enzyme, 76–608 g L^{-1} Gf-PA 6, 50 mM bicine, pH 8.0, 100 mM NaCl, 60 °C, 2 h). Reactions for individual data points were conducted in duplicate. Error bars represent the standard error of the mean.

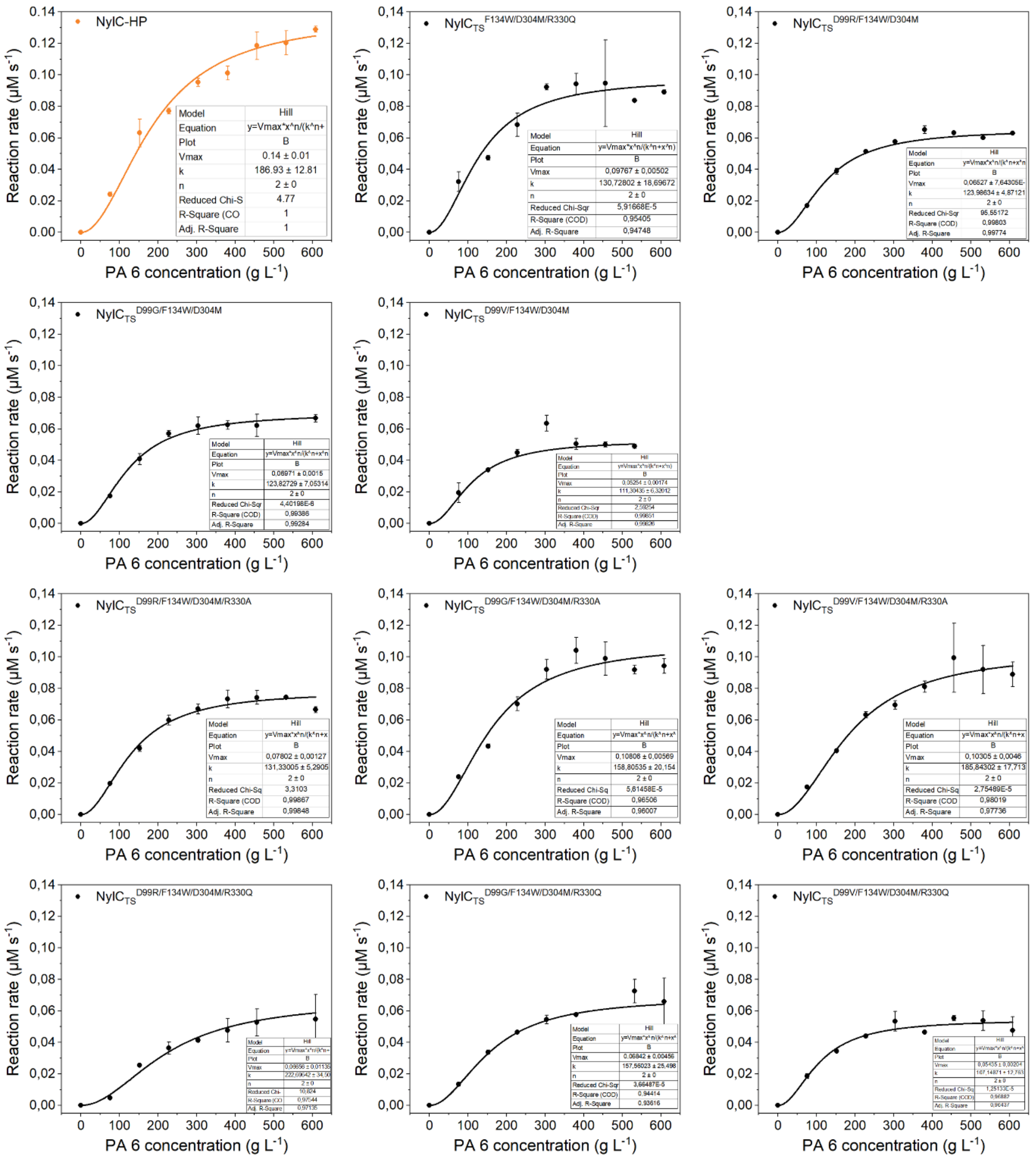

Figure S4. Conventional Michaelis-Menten kinetics of NylC$_{p2}$-TS triple- and quadruple-substitutions for PA 6.

^{Conv}MM kinetics were conducted at enzyme saturation (25 nM/0.95 mg L^{-1} enzyme, 76–608 g L^{-1} Gf-PA 6, 50 mM bicine, pH 8.0, 100 mM NaCl, 60 °C, 2 h). Reactions for individual data points were conducted in duplicate. Error bars represent the standard error of the mean.

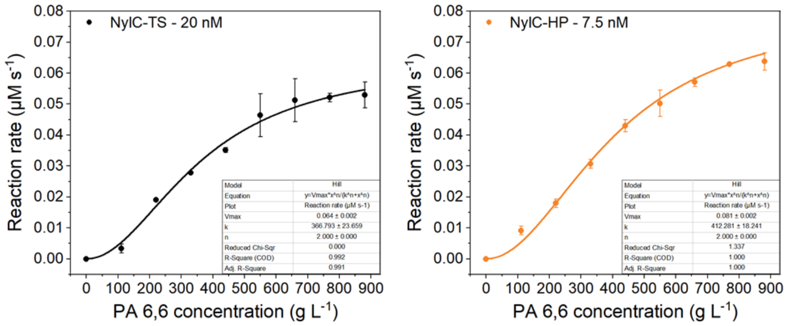

Figure S5. Conventional Michaelis-Menten kinetics of NylC$_{p2}$-TS and NylC-HP for PA 6,6.

^{Conv}MM kinetics were conducted at enzyme saturation (20 nM/0.76 mg L^{-1} or 7.5 nM/0.28 mg L^{-1} enzyme, 110–880 g L^{-1} Gf-PA 6,6, 50 mM bicine, pH 8.0, 100 mM NaCl, 60 °C, 2 h). Reactions for individual data points were conducted in duplicate. Error bars represent the standard error of the mean.

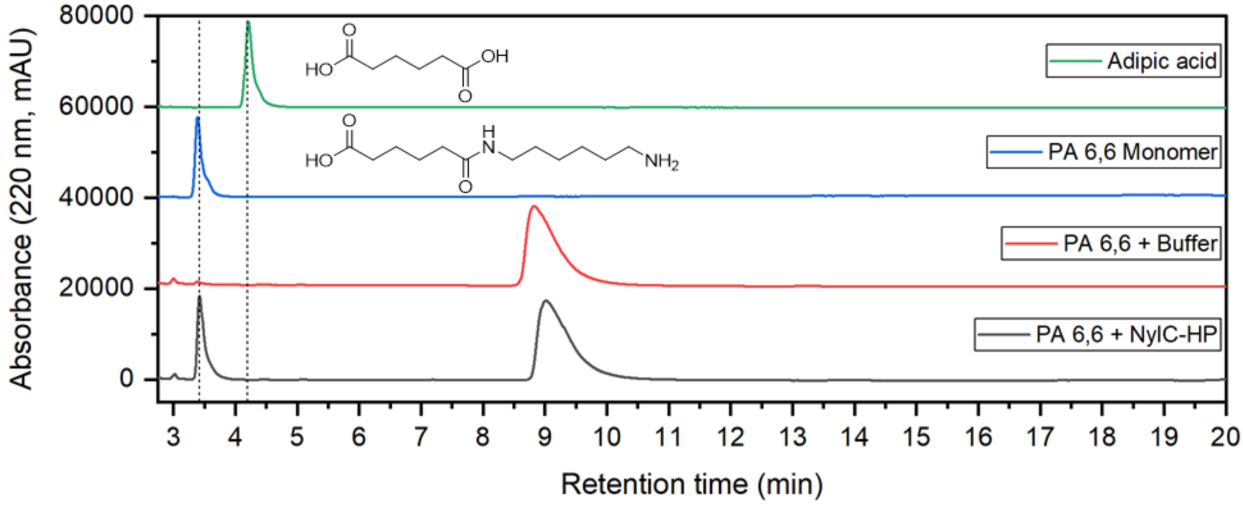

Figure S6. HPLC Chromatogram of adipic acid, PA 6,6 monomer, Gf-PA 6,6 control, and Gf-PA 6,6 degradation NylC-HP.

Adipic acid (green) could be resolved from PA 6,6 monomer (blue). In the control reaction with Gf-PA 6,6 but without enzyme, formation of an additional peak was observed. The degradation reaction (black; 50 nM/1.90 mg L^{-1} enzyme, 660 g L^{-1} Gf-PA 6, 50 mM bicine, pH 8.0, 100 mM NaCl, 60 °C, 8 h) resulted in PA 6,6 monomer as sole degradation product and no conversion of the peak extracted from Gf-PA 6,6.

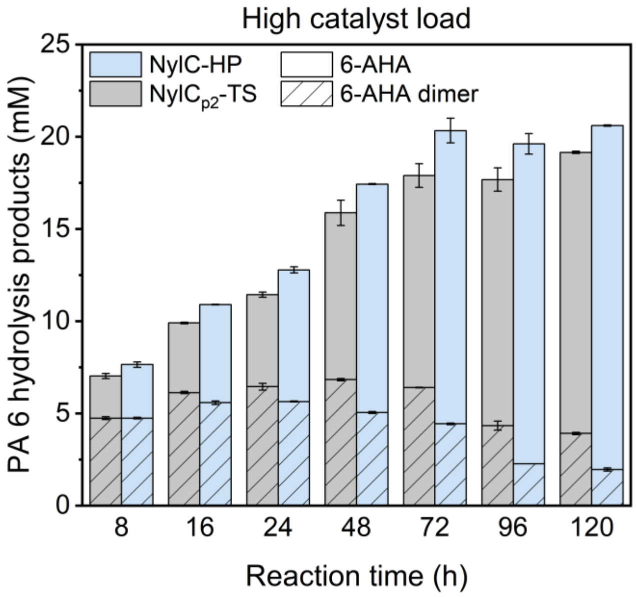

Figure S7. Time-resolved Gf-PA 6 degradation by NylC$_{p2}$-TS and NylC-HP with high catalyst load.

Reactions were conducted at substrate saturation (5 µM/190 mg L^{-1} enzyme, 456 g L^{-1} Gf-PA 6, 50 mM bicine, pH 8.0, 100 mM NaCl, 60 °C, 8–120 h).

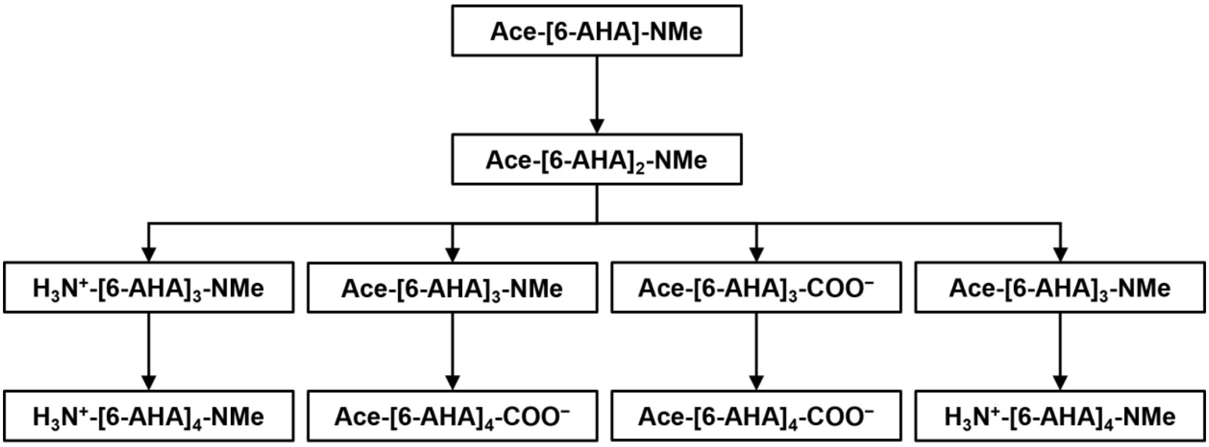

Figure S8. Incremental procedure for docking Ace-[6-AHA]$_4$-COO$^-$ and H_3N^+-[6-AHA]$_4$-NMe to the receptor.

Starting with Ace-[6-AHA]-NMe as the first fragment to be docked, both PA 6 model substrates are obtained by following each pathway characterized by a specific elongation of the fragments. In this context, 6-AHA refers to 6-aminohexanoic acid, Ace to an acetyl cap, and NMe to an N-Methyl cap.

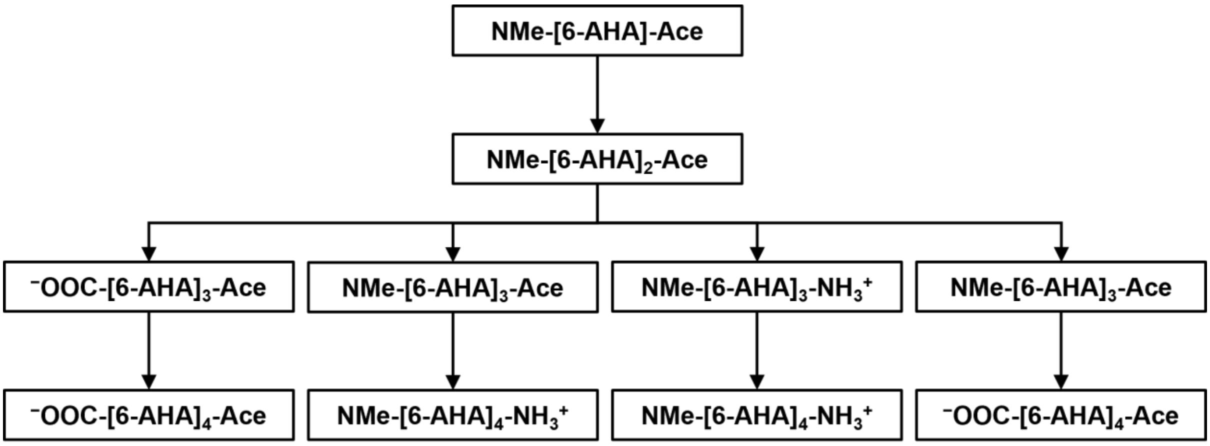

Figure S9 Incremental procedure for docking $^-$OOC-[6-AHA]$_4$-Ace and NMe-[6-AHA]$_4$-NH_3^+ to the receptor.

Starting with NMe-[6-AHA]-Ace as the first fragment to be docked, both PA 6 model substrates are obtained by following each pathway characterized by a specific elongation of the fragments. In this context, 6-AHA refers to 6-aminohexanoic acid, Ace to an acetyl cap, and NMe to an N-Methyl cap.

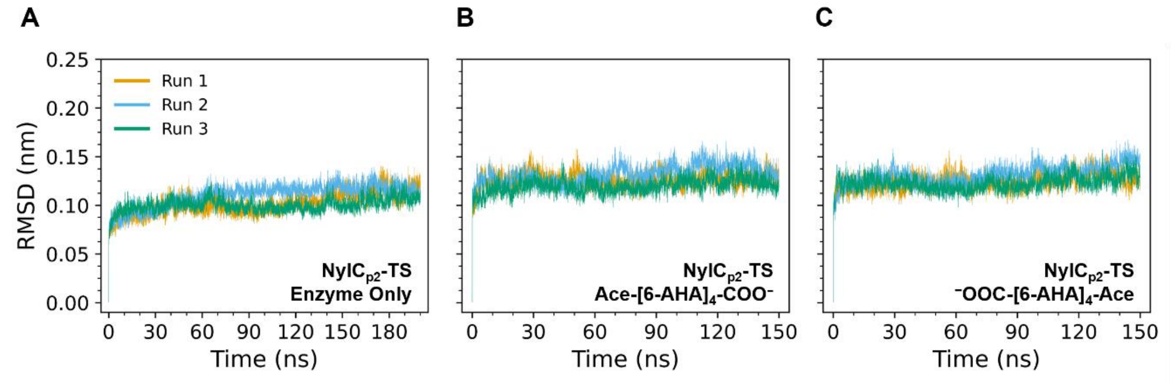

Figure S11. Root mean square deviation (RMSD) of the protein backbone of NylC$_{p2}$-TS.

RMSD after least squares fit to the backbone observed for molecular dynamics simulations of systems containing A) only the enzyme NylC$_{p2}$-TS or the enzyme-substrate complexes B) NylC$_{p2}$-TS/Ace-[6-AHA]$_4$-COO$^-$, and C) NylC$_{p2}$-TS/$^-$OOC-[6-AHA]$_4$-Ace.

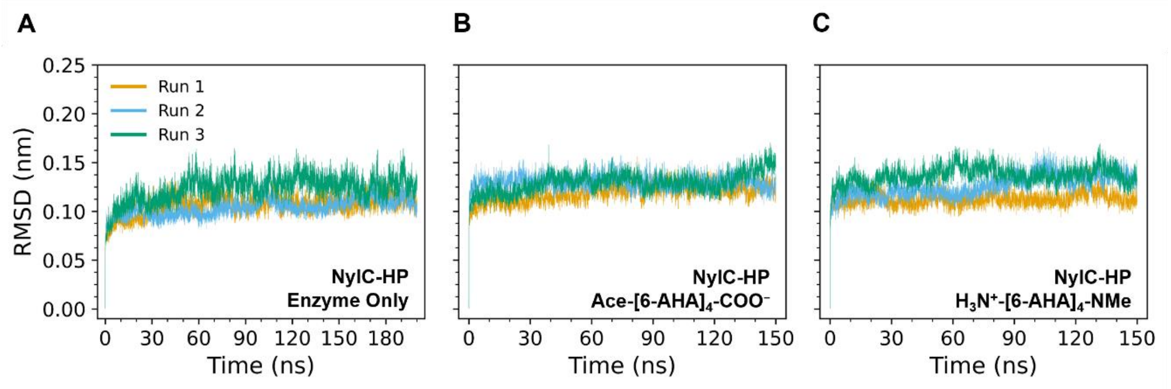

Figure S10. Root mean square deviation (RMSD) of the protein backbone of NylC$_{p2}$-HP.

RMSD after least squares fit to the backbone observed for molecular dynamics simulations of systems containing A) only the enzyme NylC$_{p2}$-TS$^{F134W/D304M/R330A}$ (NylC-HP) or the enzyme-substrate complexes B) NylC-HP/Ace-[6-AHA]$_4$-COO$^-$, and C) NylC-HP/H$_3$N$^+$-[6-AHA]$_4$-NMe.

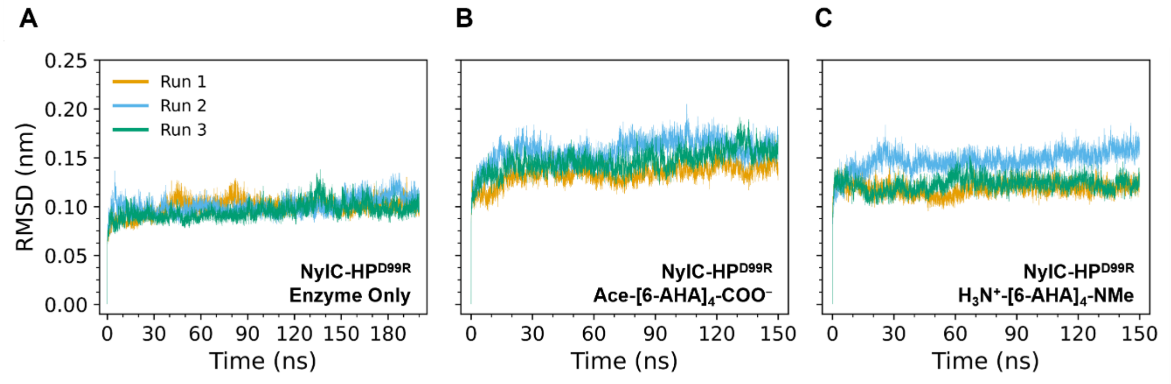

Figure S12. Root mean square deviation (RMSD) of the protein backbone of NylC-HP^{D99R}.

RMSD after least squares fit to the backbone observed for molecular dynamics simulations of systems containing A) only the enzyme NylC$_{p2}$-TS$^{D99R/F134W/D304M/R330A}$ (NylC-HP^{D99R}) or the enzyme-substrate complexes B) NylC-HP^{D99R}/Ace-[6-AHA]$_4$-COO$^-$, and C) NylC-HP^{D99R}/H$_3$N$^+$-[6-AHA]$_4$-NMe.

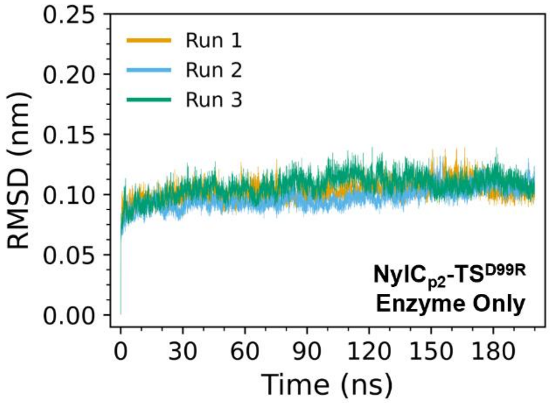

Figure S13. Root mean square deviation (RMSD) of the protein backbone of NylC$_{p2}$-TS^{D99R}.

RMSD after least squares fit to the backbone observed for molecular dynamics simulations of NylC$_{p2}$-TS^{D99R}.

Table S7. Ligand poses ranked based on their affinity score towards NylC-HP.

Calculations were performed with AutoDock Vina (version 1.2.5).[18-19] Incremental docking of PA 6 model substrates starting from Ace-[6-AHA]-NMe and NMe-[6-AHA]-Ace revealed that the substrates Ace-[6-AHA]$_4$-COO$^-$ and H_3N^+-[6-AHA]$_4$-NMe generate the most favorable poses (i.e., poses with low affinity scores and high populations). In this context, 6-AHA refers to 6-aminohexanoic acid, Ace to an acetyl cap, and NMe to an N-Methyl cap.

	**Ace-[6-AHA]$_4$-COO$^-$**		**H_3N^+-[6-AHA]$_4$-NMe**	
**Rank**	Affinity (kcal mol^{-1})	Occurrences out of 1000	Affinity (kcal mol^{-1})	Occurrences out of 1000
1	−13.84 ± 0.01	124	−13.61 ± 0.01	39
2	−13.79 ± 0.01	108	−13.60 ± 0.01	36
3	−13.71 ± 0.01	126	−13.59 ± 0.01	44
4	−13.69 ± 0.01	45	−13.59 ± 0.01	25
5	−13.67 ± 0.01	37	−13.58 ± 0.01	36
6	−13.66 ± 0.01	40	−13.58 ± 0.01	20
7	−13.65 ± 0.01	23	−13.57 ± 0.01	38
8	−13.64 ± 0.01	21	−13.54 ± 0.01	23
9	−13.62 ± 0.01	79	−13.52 ± 0.01	64
10	−13.62 ± 0.01	26	−13.51 ± 0.01	61

Table S8. Ligand poses ranked based on their affinity score towards NylC-HP^{D99R}.

Calculations were performed with AutoDock Vina (version 1.2.5).[18-19] Incremental docking of PA 6 model substrates starting from Ace-[6-AHA]-NMe and NMe-[6-AHA]-Ace revealed that the substrates Ace-[6-AHA]$_4$-COO$^-$ and H_3N^+-[6-AHA]$_4$-NMe generate the most favorable poses (i.e., poses with low affinity scores and high populations). In this context, 6-AHA refers to 6-aminohexanoic acid, Ace to an acetyl cap, and NMe to an N-Methyl cap.

	**Ace-[6-AHA]$_4$-COO$^-$**		**H_3N^+-[6-AHA]$_4$-NMe**	
**Rank**	Affinity (kcal mol^{-1})	Occurrences out of 1000	Affinity (kcal mol^{-1})	Occurrences out of 1000
1	−13.60 ± 0.01	162	−13.73 ± 0.01	28
2	−13.57 ± 0.01	140	−13.72 ± 0.01	29
3	−13.54 ± 0.02	20	−13.71 ± 0.01	24
4	−13.51 ± 0.01	113	−13.67 ± 0.01	41
5	−13.51 ± 0.01	30	−13.66 ± 0.01	44
6	−13.49 ± 0.01	37	−13.65 ± 0.01	45
7	−13.48 ± 0.01	42	−13.65 ± 0.01	36
8	−13.46 ± 0.01	27	−13.64 ± 0.01	32
9	−13.45 ± 0.01	32	−13.64 ± 0.01	28
10	−13.42 ± 0.01	21	−13.63 ± 0.01	30

Table S9. Electrostatic interactions between substrate termini and residues D99R and R330 observed during MD simulation.

Percentage of molecular dynamics (MD) simulation time with hydrogen bond ($Time_{HB}$) and salt bridge ($Time_{SB}$) existence between the carboxy-terminus of the substrate Ace-[6-AHA]$_4$-COO$^-$ and the side chain of residue R330 (chain C) of NylC$_{p2}$-TS and D99R (chain B) of NylC-HP^{D99R}. MD simulations were performed in triplicates for 150 ns each, with the first 20 ns not considered for analysis.

**System**	**Residue**	**Run**	**$Time_{HB}$ (%)**	**$Time_{SB}$ (%)**
NylC$_{p2}$-TS / $^-$OOC-[6-AHA]$_4$-Ace	R330	1	79	79
		2	87	87
		3	34	29
		Mean	67 ± 16	65 ± 18
NylC-HP^{D99R} / Ace-[6-AHA]$_4$-COO$^-$	D99R	1	89	89
		2	98	98
		3	2	2

Table S10. Change in interaction energy upon H-bond formation between substrate termini and residues D99R and R330 observed during MD simulation.

Mean interaction energy and standard deviation of the substrate Ace-[6-AHA]$_4$-COO$^-$ and the enzymes NylC$_{p2}$-TS and NylC-HP^{D99R} with established (E_{HB}) and not established ($E_{no\ HB}$) hydrogen bonds (H-bonds) between the side chain of specified residues (R330 (chain C) and D99R (chain B)) of the enzymes and the carboxy-terminus of the substrate observed during molecular dynamics (MD) simulation. MD simulations were performed in triplicates for 150 ns each, with the first 20 ns not considered for analysis. As H-bond formation for MD simulation run 3 of the system NylC-HP^{D99R}/Ace-[6-AHA]$_4$-COO$^-$ could only be observed for 2 % of the simulation time, this value was not considered for further analysis.

System	Residue	Run	E_{HB} (kJ mol^{-1})	$E_{no\ HB}$ / (kJ mol^{-1})	ΔE / (kJ mol^{-1})
NylC$_{p2}$-TS / $^-$OOC-[6-AHA]$_4$-Ace	R330	1	−429 ± 40	−337 ± 38	−92 ± 55
		2	−494 ± 35	−393 ± 27	−101 ± 44
		3	−372 ± 57	−271 ± 27	−101 ± 63
		Mean			−98 ± 18
NylC-HP^{D99R} / Ace-[6-AHA]$_4$-COO$^-$	D99R	1	−384 ± 33	−303 ± 41	−81 ± 53
		2	−421 ± 34	−319 ± 39	−102 ± 52
		(3	−379 ± 45	−377 ± 49	−2 ± 66)
		Mean			−92 ± 26

Table S11. Electrostatic interactions between residues D191-R330 and D99R-D304 observed during MD simulation.

Percentage of molecular dynamics (MD) simulation time with hydrogen bond ($Time_{HB}$) and salt bridge ($Time_{SB}$) existence between the side chains of specified residues (i.e., D191 (chain A) and R330 (chain C) as well as D99R (chain B) and D304 (chain A)) for the enzymes NylC$_{p2}$-TS and NylC$_{p2}$-TS^{D99R}. MD simulations were performed in triplicates for 200 ns each, with the first 20 ns not considered for analysis.

System	Residues	Run	$Time_{HB}$ (%)	$Time_{SB}$ / (%)
NylC$_{p2}$-TS	D191-R330	1	30	28
		2	29	28
		3	9	8
NylC$_{p2}$-TS^{D99R}	D191-R330	1	46	45
		2	11	10
		3	27	25
	D99R-D304	1	20	20
		2	46	46
		3	51	51

Table S12. Time of residue F134 being in distinct conformations for different systems containing NylC$_{p2}$-TS.

Percentage of molecular dynamics (MD) simulation time of residue F134 (chain B) being in conformation 1 (Time$_{Conf. 1}$) and conformation 2 (Time$_{Conf. 2}$) for different systems containing NylC$_{p2}$-TS. Conformation 1 is characterized by a vertically oriented side chain of F134, which prevents intramolecular interactions of the substrate. Conformation 2 is characterized by a horizontal alignment of the side chain of F134. MD simulations were performed in triplicates for 150 ns (with substrate) and 200 ns (without substrate) each, with the first 20 ns not considered for analysis.

Substrate	Run	Time$_{Conf. 1}$ (%)	Time$_{Conf. 2}$ (%)
	1	95.7	4.3
Ace-[6-AHA]$_4$-COO$^-$	2	27.3	72.7
	3	100.0	0.0
	1	100.0	0.0
$^-$OOC-[6-AHA]$_4$-Ace	2	100.0	0.0
	3	12.0	88.0
	1	97.6	2.4
No substrate	2	96.8	3.2
	3	98.1	1.9

Table S13. Time of residue F134W being in distinct conformations for different systems containing NylC$_{p2}$-HP.

Percentage of molecular dynamics (MD) simulation time of residue F134W (chain B) being in conformation 1 (Time$_{Conf. 1}$) and conformation 2 (Time$_{Conf. 2}$) for different systems containing NylC$_{p2}$-TS$^{F134W/D304M/R330A}$ (NylC-HP). Conformation 1 is characterized by a vertically oriented side chain of F134W, which prevents intramolecular interactions and (optionally) fixes the amide bond to be cleaved by H-bonding close to the active residue T267. Conformation 2 is characterized by a horizontal alignment of the side chain of F134W. MD simulations were performed in triplicates for 150 ns (with substrate) and 200 ns (enzyme only) each, with the first 20 ns not considered for analysis.

Substrate	Run	Time$_{Conf. 1}$ (%)	Time$_{Conf. 2}$ (%)
	1	0.0	100.0
Ace-[6-AHA]$_4$-COO$^-$	2	100.0	0.0
	3	34.7	65.3
	1	89.4	10.6
H_3N^+-[6-AHA]$_4$-NMe	2	100.0	0.0
	3	100.0	0.0
	1	99.7	0.3
No substrate	2	100.0	0.0
	3	100.0	0.0

Table S14. Time of residue F134W being in distinct conformations for different systems containing NylC-HP^{D99R}.

Percentage of molecular dynamics (MD) simulation time of residue F134W (chain B) being in conformation 1 ($Time_{Conf. 1}$) and conformation 2 ($Time_{Conf. 2}$) for different systems containing NylC$_{p2}$-TS$^{D99R/F134W/D304M/R330A}$ (NylC-HP^{D99R}). Conformation 1 is characterized by a vertically oriented side chain of F134W, which prevents intramolecular interactions and (optionally) fixes the amide bond to be cleaved by H-bonding close to the active residue T267. Conformation 2 is characterized by a horizontal alignment of the side chain of F134W. MD simulations were performed in triplicates for 150 ns (with substrate) and 200 ns (enzyme only) each, with the first 20 ns not considered for analysis.

Substrate	Run	$Time_{Conf. 1}$ (%)	$Time_{Conf. 2}$ (%)
	1	100.0	0.0
Ace-[6-AHA]$_4$-COO$^-$	2	79.7	20.3
	3	100.0	0.0
	1	100.0	0.0
H_3N^+-[6-AHA]$_4$-NMe	2	100.0	0.0
	3	100.0	0.0
	1	100.0	0.0
No substrate	2	99.6	0.4
	3	100.0	0.0

Table S15. Percentage of molecular dynamics (MD) simulation time of residue F134 (chain B) being in conformation 1 ($Time_{Conf. 1}$) and conformation 2 ($Time_{Conf. 2}$) for different systems containing NylC$_{p2}$-TS^{D99R}.

Conformation 1 is characterized by a vertically oriented side chain of F134. Conformation 2 is characterized by a horizontal alignment of the side chain of F134. MD simulations were performed in triplicates for 200 ns each, with the first 20 ns not considered for analysis.

Substrate	Run	$Time_{Conf. 1}$ (%)	$Time_{Conf. 2}$ (%)
	1	99.4	0.6
No substrate	2	93.5	6.5
	3	35.6	64.4

Table S16. H-bonds between the substrate and residue at position 134 observed during MD simulation.

Percentage of molecular dynamics (MD) simulation time with hydrogen bond existence between the side chain of residue at position 134 (chain B) for different enzymes (i.e., $NylC_{p2}$-TS, NylC-HP, and $NylC\text{-}HP^{D99R}$) and the amide bond to be cleaved of various substrates (i.e., Ace-$[6\text{-AHA}]_4$-COO^-, ^-OOC-$[6\text{-AHA}]_4$-Ace, and H_3N^+-$[6\text{-AHA}]_4$-NMe). MD simulations were performed in triplicates for 200 ns each, with the first 20 ns not considered for analysis.

	System with X = $[6\text{-AHA}]_4$					
**Enzyme**	**$NylC_{p2}$-TS**		**NylC-HP**		**$NylC\text{-}HP^{D99R}$**	
**Run**	Ace-X-COO^-	^-OOC-X-Ace	Ace-X-COO^-	H_3N^+-X-NMe	Ace-X-COO^-	H_3N^+-X-NMe
1	0	0	0	25	1	16
2	0	0	6	8	0	0
3	0	0	0	5	0	12

Table S17. List of primers

Primer name	Primer sequence
pET21a_NylC$_{p2}$-TS_SSM V18_fwd	GTGGTATTGCA**NNK**GATCCGGCACCGCGTCTG
pET21a_NylC$_{p2}$-TS_SSM V18_rev	CATCAATATCGGTCAGTGCATGAACCGGTGTGGTATTGG
pET21a_NylC$_{p2}$-TS_SSM L24_fwd	CACCGCGT**NNK**GCAGGTCCGCCTGTTTTTG
pET21a_NylC$_{p2}$-TS_SSM L24_rev	CCGGATCAACTGCAATACCACCATCAATATCGGTCAGTG
pET21a_NylC$_{p2}$-TS_SSM P27_fwd	GTGGTCCGGGTAATGCTGCATTC
pET21a_NylC$_{p2}$-TS_SSM P27_rev	CAAAAACAGG**MNN**ACCTGCCAG
pET21a_NylC$_{p2}$-TS_SSM F38_fwd	CGGTTCGTAGCACCGGTCGTG
pET21a_NylC$_{p2}$-TS_SSM F38_rev	GTGCCAGATC**MNN**TGCAGCATTACCC
pET21a_NylC$_{p2}$-TS_SSM A91_fwd	CACGTGGTGGT**NNK**GTTGGTCTGAGC
pET21a_NylC$_{p2}$-TS_SSM A91_rev	CATCAACTGCGGTACGTGCGCCTGCC
pET21a_NylC$_{p2}$-TS_SSM Y98_fwd	CAGTTGGTCTGAGCGGTGGT**NNK**GATTTTAATCATGCAATTTGCC
pET21a_NylC$_{p2}$-TS_SSM Y98_rev	CACCACCACGTGCATCAACTGCGGTACG
pET21a_NylC$_{p2}$-TS_SSM D99_fwd	GTCTGAGCGGTGGTTAT**NNK**TTTAATCATGCAATTTGC
pET21a_NylC$_{p2}$-TS_SSM D99_rev	CAACTGCACCACCACGTGCATCAACTG
pET21a_NylC$_{p2}$-TS_SSM F100_fwd	GCAGGCGGTGCAGGTTATGG
pET21a_NylC$_{p2}$-TS_SSM F100_rev	CAGGCAAATTGCATGATT**MNN**ATCATAACCACCGCTC
pET21a_NylC$_{p2}$-TS_SSM G111_fwd	GCGGTGCA**NNK**TATGGTCTGGAAGCCGG
pET21a_NylC$_{p2}$-TS_SSM G111_rev	CTGCCAGGCAAATTGCATGATTAAAATCATAACCACCGC
pET21a_NylC$_{p2}$-TS_SSM F134_fwd	GAATATCGTACCGGT**NNK**GCAGAACTGCAGCTGG
pET21a_NylC$_{p2}$-TS_SSM F134_rev	CAGACGTTCCAGCAGTGCACCACTAACACC
pET21a_NylC$_{p2}$-TS_SSM L137_fwd	CGGTTTTGCAGAA**NNK**CAGCTGGTTAGCAGC
pET21a_NylC$_{p2}$-TS_SSM L137_rev	TACGATATTCCAGACGTTCCAGCAGTGCACCAC
pET21a_NylC$_{p2}$-TS_SSM L139_fwd	GTTTTGCAGAACTGCAG**NNK**GTTAGCAGCGC
pET21a_NylC$_{p2}$-TS_SSM L139_rev	CGGTACGATATTCCAGACGTTCCAGCAGTGCACC
pET21a_NylC$_{p2}$-TS_SSM V144_fwd	GTTAGCAGCGCA**NNK**ATCTATGATTTTTCAGCAC
pET21a_NylC$_{p2}$-TS_SSM V144_rev	CAGCTGCAGTTCTGCAAAACCGGTAC
pET21a_NylC$_{p2}$-TS_SSM Y146_fwd	GTTAGCAGCGCAGTTATC**NNK**GATTTTTCAGCACGTTCAAC
pET21a_NylC$_{p2}$-TS_SSM Y146_rev	CAGCTGCAGTTCTGCAAAACCGGTACGATATTCC
pET21a_NylC$_{p2}$-TS_SSM A160_fwd	GTTTATCCTGATAAA**NNK**CTGGGTCGTGCAGCACTGG
pET21a_NylC$_{p2}$-TS_SSM A160_rev	TGCGGTTGAACGTGCTGAAAAATCATAGATAACTGCGCTG
pET21a_NylC$_{p2}$-TS_SSM D209_fwd	CAGTTGTTGTTCCGAATCCGGTTGGTG
pET21a_NylC$_{p2}$-TS_SSM D209_rev	CCAGAATACGAAC**MNN**ACCCAGACGACG
pET21a_NylC$_{p2}$-TS_SSM F301_fwd	GTGATACCCTGTTTGCAGTTACCACCG
pET21a_NylC$_{p2}$-TS_SSM F301_rev	CATCCATATCTGTATG**MNN**CGGCTGAATGCCACG
pET21a_NylC$_{p2}$-TS_SSM D304_fwd	CCGTTTCATACA**NNK**ATGGATGGTGATACCCTGTTTGC
pET21a_NylC$_{p2}$-TS_SSM D304_rev	CTGAATGCCACGATGCATGCTGCTATGAAC
pET21a_NylC$_{p2}$-TS_SSM M305_fwd	CAGTTACCACCGATGAAATTGATCTGCCG

pET21a_NylC$_{p2}$-TS_SSM M305_rev	CAAACAGGGTATCACCATC**MNN**ATCTGTATGAAACGG
pET21a_NylC$_{p2}$-TS_SSM R330_fwd	GTAGCAGCCGTGGT**NNK**CTGAGCGTTAATGCAAC
pET21a_NylC$_{p2}$-TS_SSM R330_rev	CCGGTGTTGTCGGCAGATCAATTTCATCGGTGG
pET21a_NylC$_{p2}$-TS_SDM D99G_fwd	GGTTAT**GGT**TTTAATCATGCAAT
pET21a_NylC$_{p2}$-TS_SDM D99G_rev	ATTAAA**ACC**ATAACCACCGCTCAG
pET21a_NylC$_{p2}$-TS_SSM D99V_fwd	GGTTAT**GTT**TTTAATCATGCAAT
pET21a_NylC$_{p2}$-TS_SSM D99V_rev	ATTAAA**AAC**ATAACCACCGCTCAG
pET21a_NylC$_{p2}$-TS_SDM D99R_fwd	GCAGTTGGTCTGAGCGGTGGTTAT**CGC**TTTAATCATGCAATTTGC
pET21a_NylC$_{p2}$-TS_SDM D99R_rev	ACCACCACGTGCATCAACTGCGGTACGTG
pET21a_NylC$_{p2}$-TS_SDM F134W_fwd	GAATATCGTACCGGT**TGG**GCAGAACTGCAGCTGG
pET21a_NylC$_{p2}$-TS_SDM F134W_rev	CAGACGTTCCAGCAGTGCACCACTAACACC
pET21a_NylC$_{p2}$-TS_SDM D304M_fwd	CCGTTTCATACA**ATG**ATGGATGGTGATACCCTGTTTGC
pET21a_NylC$_{p2}$-TS_SDM D304_rev	CTGAATGCCACGATGCATGCTGCTATGAAC
pET21a_NylC$_{p2}$-TS_SSM R330A_fwd	GTAGCAGCCGTGGT**GCG**CTGAGCGTTAATGCAAC
pET21a_NylC$_{p2}$-TS_SDM R330_rev	CCGGTGTTGTCGGCAGATCAATTTCATCGGTGGTAAC
pET21a_NylC$_{p2}$-TS_SDM R330Q_fwd	CGTGGT**CAG**CTGAGCGTTAATGC
pET21a_NylC$_{p2}$-TS_SDM R330Q_rev	GCTCAG**CTG**ACCACGGCTGCTACC

REFERENCES

[1] S. Negoro, N. Shibata, Y. Tanaka, K. Yasuhira, H. Shibata, H. Hashimoto, Y.-H. Lee, S. Oshima, R. Santa, S. Oshima, K. Mochiji, Y. Goto, T. Ikegami, K. Nagai, D.-I. Kato, M. Takeo and Y. Higuchi, *J. Biol. Chem.* **2012**, *287*, 5079-5090.
[2] H. Puetz, C. Janknecht, F. Contreras, M. Vorobii, T. Kurkina and U. Schwaneberg, *ACS Sustainable Chem. Eng.* **2023**, *11*, 15513−15522.
[3] M. M. Bradford, *Anal. Biochem.* **1976**, *72*, 248-254.
[4] S. Matsuoka, *Applications to Polymers and Plastics*, **2002**, p.
[5] L. Zeußel, P. Mai, S. Sharma, A. Schober, S. Ren and S. Singh, *ChemistrySelect* **2021**, *6*, 6834-6840.
[6] S. Negoro, N. Shibata, Y.-H. Lee, I. Takehara, R. Kinugasa, K. Nagai, Y. Tanaka, D.-I. Kato, M. Takeo, Y. Goto and Y. Higuchi, *Sci. Rep.* **2018**, *8*.
[7] H. M. Berman, J. Westbrook, Z. Feng, G. Gilliland, T. N. Bhat, H. Weissig, I. N. Shindyalov and P. E. Bourne, *Nucleic Acids Res.* **2000**, *28*, 235-242.
[8] A. Šali and T. L. Blundell, *J. Mol. Biol.* **1993**, *234*, 779-815.
[9] H. J. C. Berendsen, D. van der Spoel and R. van Drunen, *Comput. Phys. Commun.* **1995**, *91*, 43-56.
[10] D. Van Der Spoel, E. Lindahl, B. Hess, G. Groenhof, A. E. Mark and H. J. C. Berendsen, *J. Comput. Chem.* **2005**, *26*, 1701-1718.
[11] M. J. Abraham, T. Murtola, R. Schulz, S. Páll, J. C. Smith, B. Hess and E. Lindahl, *SoftwareX* **2015**, *1-2*, 19-25.
[12] M. R. Machado and S. Pantano, *J. Chem. Theory Comput.* **2020**, *16*, 1367-1372.
[13] P. A. Ravindranath, S. Forli, D. S. Goodsell, A. J. Olson and M. F. Sanner, *PLoS Comput. Biol.* **2015**, *11*, e1004586.
[14] M. D. Hanwell, D. E. Curtis, D. C. Lonie, T. Vandermeersch, E. Zurek and G. R. Hutchison, *J. Cheminf.* **2012**, *4*, 17.
[15] A. Dhanik, J. S. McMurray and L. E. Kavraki, *BMC Struct. Biol.* **2013**, *13*, S11.
[16] A. Dhanik, J. S. McMurray and L. Kavraki, *IEEE Int Conf Bioinformatics Biomed* **2012**, pp. 48-55.
[17] D. Devaurs, D. A. Antunes, S. Hall-Swan, N. Mitchell, M. Moll, G. Lizée and L. E. Kavraki, *BMC Mol. Cell Biol.* **2019**, *20*, 42.
[18] J. Eberhardt, D. Santos-Martins, A. F. Tillack and S. Forli, *J. Chem. Inf. Model.* **2021**, *61*, 3891-3898.
[19] O. Trott and A. J. Olson, *J. Comput. Chem.* **2010**, *31*, 455-461.
[20] W. Yu, X. He, K. Vanommeslaeghe and A. D. MacKerell Jr, *J. Comput. Chem.* **2012**, *33*, 2451-2468.
[21] K. Vanommeslaeghe, E. Hatcher, C. Acharya, S. Kundu, S. Zhong, J. Shim, E. Darian, O. Guvench, P. Lopes, I. Vorobyov and A. D. Mackerell Jr, *J. Comput. Chem.* **2010**, *31*, 671-690.
[22] S. F. Altschul, W. Gish, W. Miller, E. W. Myers and D. J. Lipman, *J. Mol. Biol.* **1990**, *215*, 403-410.
[23] F. Sievers, A. Wilm, D. Dineen, T. J. Gibson, K. Karplus, W. Li, R. Lopez, H. McWilliam, M. Remmert, J. Söding, J. D. Thompson and D. G. Higgins, *Mol. Syst. Biol.* **2011**, *7*, 539.
[24] M. Goujon, H. McWilliam, W. Li, F. Valentin, S. Squizzato, J. Paern and R. Lopez, *Nucleic Acids Res.* **2010**, *38*, W695-W699.
[25] F. Madeira, N. Madhusoodanan, J. Lee, A. Eusebi, A. Niewielska, A. R. N. Tivey, R. Lopez and S. Butcher, *Nucleic Acids Res.* **2024**, W521-W525.
[26] T. D. Schneider and R. M. Stephens, *Nucleic Acids Res.* **1990**, *18*, 6097-6100.
